# Neural Pathway for Gut Feelings: Vagal Interoceptive Feedback From the Gastrointestinal Tract Is a Critical Modulator of Anxiety-like Behavior

**DOI:** 10.1016/j.biopsych.2022.04.020

**Published:** 2022-05-18

**Authors:** Jean-Philippe Krieger, Mohammed Asker, Pauline van der Velden, Stina Börchers, Jennifer E. Richard, Ivana Maric, Francesco Longo, Arashdeep Singh, Guillaume de Lartigue, Karolina P. Skibicka

**Affiliations:** Institute of Neuroscience and Physiology, University of Gothenburg; Wallenberg Centre for Molecular and Translational Medicine, Gothenburg, Sweden; Institute of Neuroscience and Physiology, University of Gothenburg; Wallenberg Centre for Molecular and Translational Medicine, Gothenburg, Sweden; Institute of Neuroscience and Physiology, University of Gothenburg; Institute of Neuroscience and Physiology, University of Gothenburg: Wallenberg Centre for Molecular and Translational Medicine, Gothenburg, Sweden; Institute of Neuroscience and Physiology, University of Gothenburg; Wallenberg Centre for Molecular and Translational Medicine, Gothenburg, Sweden; Institute of Neuroscience and Physiology, University of Gothenburg; Wallenberg Centre for Molecular and Translational Medicine, Gothenburg, Sweden; Institute of Neuroscience and Physiology, University of Gothenburg; Wallenberg Centre for Molecular and Translational Medicine, Gothenburg, Sweden; Department of Pharmacodynamics, College of Pharmacy; Center for Integrative Cardiovascular and Metabolic Disease, University of Florida, Gainesville, Florida; Department of Pharmacodynamics, College of Pharmacy; Center for Integrative Cardiovascular and Metabolic Disease, University of Florida, Gainesville, Florida; Institute of Neuroscience and Physiology, University of Gothenburg; Wallenberg Centre for Molecular and Translational Medicine, Gothenburg, Sweden; Nutritional Sciences, College of Health and Human Development, Pennsylvania State University, State College, Pennsylvania

## Abstract

**BACKGROUND:**

Anxiety disorders are associated with an altered perception of the body’s internal state. Therefore, understanding the neuronal basis of interoception can foster novel anxiety therapies. In rodents, the feeding status bidirectionally modulates anxiety-like behavior but how the sensing of gastrointestinal state affects anxiety remains unclear.

**METHODS:**

We combined chemogenetics, neuropharmacology, and behavioral approaches in male and female rats to test whether vagal afferents terminating in the gastrointestinal tract mediate feeding-induced tuning of anxiety. Using saporin-based lesions and transcriptomics, we investigated the chronic impact of this gut-brain circuit on anxiety-like behavior.

**RESULTS:**

Both feeding and selective chemogenetic activation of gut-innervating vagal afferents increased anxiety-like behavior. Conversely, chemogenetic inhibition blocked the increase in anxiety-like behavior induced by feeding. Using a selective saporin-based lesion, we demonstrate that the loss of gut-innervating vagal afferent signaling chronically reduces anxiety-like behavior in male rats but not in female rats. We next identify a vagal circuit that connects the gut to the central nucleus of the amygdala, using anterograde transsynaptic tracing from the nodose ganglia. Lesion of this gut-brain vagal circuit modulated the central amygdala transcriptome in both sexes but selectively affected a network of GABA (gamma-aminobutyric acid)-related genes only in males, suggesting a potentiation of inhibitory control. Blocking GABAergic signaling in the central amygdala re-established normal anxiety levels in male rats.

**CONCLUSIONS:**

Vagal sensory signals from the gastrointestinal tract are critical for baseline and feeding-induced tuning of anxiety via the central amygdala in rats. Our results suggest vagal gut-brain signaling as a target to normalize interoception in anxiety disorders.

Anxiety disorders affect 300 million individuals per year worldwide (1). Despite this immense burden, the underlying mechanisms implicated in the etiology of anxiety disorders remain elusive. Remarkably, patients with anxiety disorders generally present hypersensitivity and hypervigilance to somatic sensations, accompanied by a danger-like interpretation of these sensations (2). Thus, a dysfunctional interoception (the brain’s ability to adaptively perceive the body’s internal states) is recognized as a component of anxiety disorders (3), and anxiety therapies increasingly target interoceptive physiology (e.g., via the use of pharmacological agents or cognitive behavioral therapy) (4). Hence, identifying the neural mechanisms of interoception and how they become dysfunctional in anxiety disorders is crucial for the development of anxiety therapies and overall, to improve mental health.

A striking example of the interoceptive modulation of anxiety can be observed in rodents following changes in metabolic states sensed by the gastrointestinal tract. Mice exhibit less anxiety-like behavior toward the end of the light phase (i.e., shortly before mice consume most of their daily calories) than in the beginning of the light phase (5). In addition, periods of fasting or calorie restriction reduce anxiety-like behavior compared with ad libitum feeding (6-11). This tuning of anxiety-like behavior necessitates that feeding-induced metabolic signals reach the brain to induce relevant behavioral output. The identity of the underlying gut-brain circuits remains unknown.

Vagal afferent neurons are ideally positioned to mediate the metabolic tuning of anxiety. Gut-innervating vagal neurons form peripheral terminals that detect mechanical (i.e., distension, smooth muscle tone) or chemical (i.e., gut hormone release) signals in the gastrointestinal tract (12). These signals are conveyed to the brain via the central terminals of vagal afferents within the caudal nucleus tractus solitarii and reach anxiety-controlling brain areas such as the bed nucleus of the stria terminalis (BNST) and the central nucleus of the amygdala (CeA) via multisynaptic circuits [see (13) for review]. Finally, although vagal afferents are primarily described for their role in satiation, emerging evidence indicates that the gut-brain axis modulates a wide array of behaviors, including reward (14), memory (15), and anxiety: subdiaphragmatic vagal deafferentation (SDA) reduces innate anxiety (16), and vagotomy abolishes the anxiety effects of gut microbes and intestinal probiotics in rodents (17-19).

Here, we first test whether vagal afferent neurons, specifically those terminating into the gastrointestinal tract, mediate the metabolic tuning of anxiety-like behavior in rats. For this, we adapted a novel 2-vector strategy (14) for the chemogenetic manipulation of vagal afferents that innervate the stomach and the duodenum in rats and therefore overcame the lack of selectivity of previously established vagal surgeries (20). We next asked whether a chronic interruption of vagal feedback from the gastrointestinal tract to the brain leads to sustained changes in anxiety-like behavior, independent of food intake. Finally, we used transsynaptic tracing and RNA sequencing to investigate the contribution of 2 anxiety-controlling brain areas (CeA and BNST) to the vagal modulation of anxiety-like behavior.

## METHODS AND MATERIALS

### Animals and Housing

Male and female Sprague Dawley (*n* = 264, Charles River) and male Wistar (*n* = 3, Envigo) rats had ad libitum access to water and standard laboratory chow, unless otherwise noted. All procedures were approved by the Swedish Agriculture Ministry and University of Gothenburg animal ethics board (Approvals #1/19 and 1/21) or Institutional Animal Care & Use Committee (Protocol #202110305). Additional housing information is found in Supplemental Methods.

### Surgeries

#### Gastrointestinal Injections.

The viral solution (AAVrg-pENN.AAV.hSyn.HI.eGFP-Cre.WPRE.SV40, 1.3 × 10^13^ GC/mL, Addgene 105540-AAVrg) was loaded into borosilicate capillaries (Warner Instruments) and injected below the gastrointestinal serosa. A total of 10 μL (stomach 7 μL, duodenum 3 μL) was delivered to 36 predefined sites (see Supplemental Methods and Figure S2G).

#### Nodose Ganglia Injections.

The viral solutions (AAV5-hSyn-DIO-hm3D(Gq)-mCherry, 1.0 × 10^13^ GC/mL, Addgene 44361; AAV5-hSyn-DIO-hm4D(Gi)-mCherry, 1.4 × 10^13^ GC/mL, Addgene 44362; AAV5-hSyn-DIO-mCherry, 1.2 × 10^13^ GC/mL, Addgene 50459; H129-CMV-mCherry, 9.55 × 10^8^ GC/mL, NIH Center for Neuroanatomy with Neurotropic Virus, Catalog No. 373; H129-CMV-EGFP, 1.0 × 10^9^ GC/mL, NIH Center for Neuroanatomy with Neurotropic Virus, Catalog No. 772) or saporin toxins (saporin toxin conjugated to cholecystokinin [CCK-SAP], IT31 and Blank-SAP, IT21, 250 ng/μL, Advanced Targeting Systems) were loaded into borosilicate capillaries (Warner Instruments). The capillaries were inserted into the nodose ganglia between the superior laryn-geal nerve and pharyngeal nerve. A total of 1.5 μL of viral/toxin solution was delivered into each nodose ganglion.

### Assessment of Anxiety-like Behavior

#### General Research Design.

Six separate cohorts were assessed for anxiety-like behavior using 4 complementary tests, conducted in the following order: elevated plus maze, open field, acoustic startle reflex, and food neophobia. Cohorts and tests are further described in Supplemental Methods.

### Graphs and Statistics

All graphs represent mean ± standard error of the mean and were generated with R (version 3.6.1-4.4.1). Tests *F* and *p* values are reported in the figure legends. Statistical approach to sex-specific effects is described in Supplemental Methods.

## RESULTS

### The Feeding Status Modulates Anxiety-like Behavior

**Figure 1.**
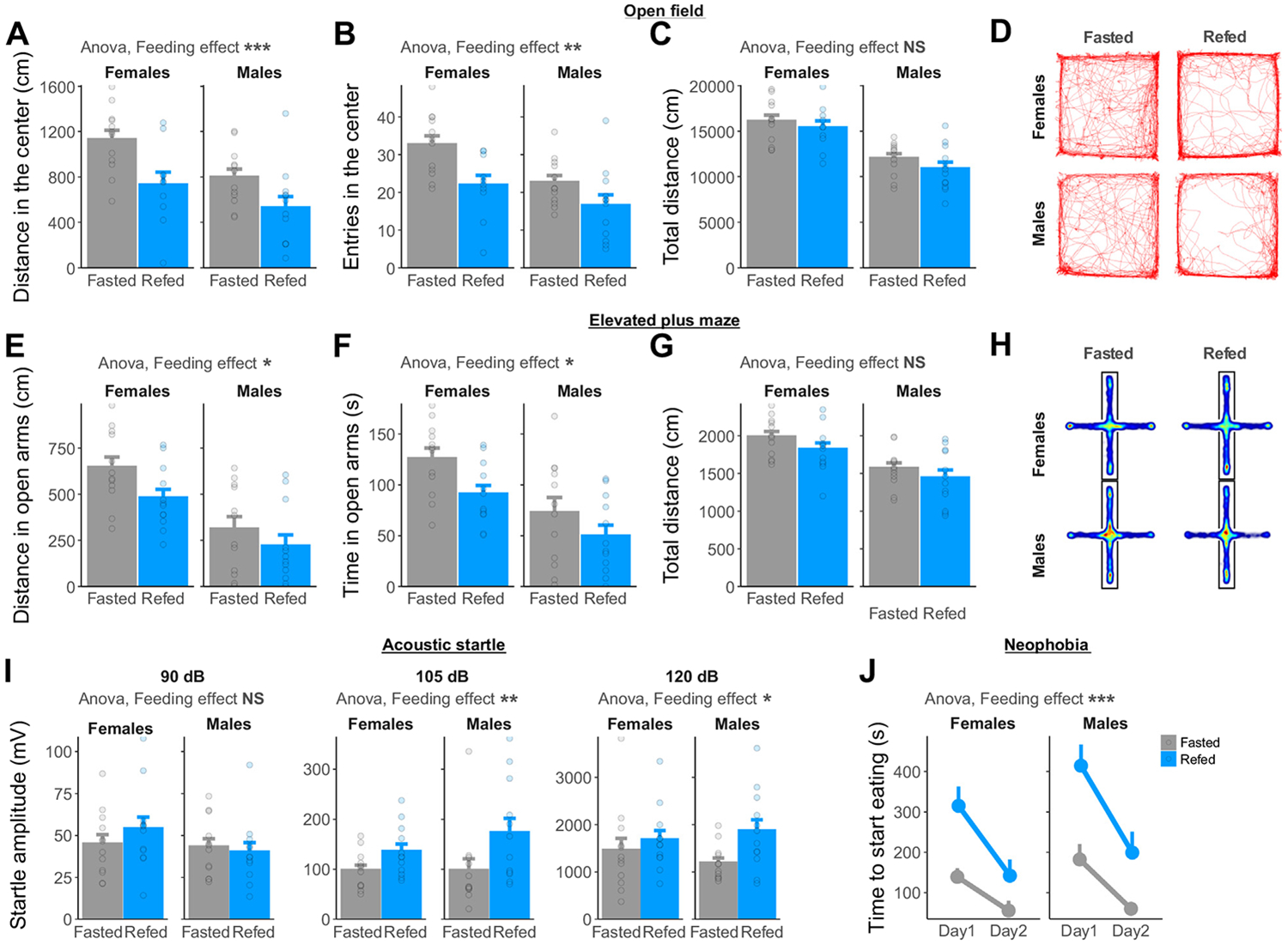
Refeeding increases anxiety-like behavior in male and female rats compared with fasting. **(A)** Distance (ANOVA, *F*_1,43_ = 12.79, *p* = .0009) and **(B)** number of entries (ANOVA, *F*_1,43_ = 11.38, *p* = .002) in the center of an open field decrease upon refeeding in male and female rats. **(C)** The total distance moved in an open field is not significantly changed by the feeding status (ANOVA, *F*_1,43_ = 1.90, *p* = .18). **(D)** Representative traces of fasted or refed rats during a 30-minute open field test. **(E)** Distance (ANOVA, *F*_1,45_ = 4.70, *p* = .035) and **(F)** time (ANOVA, *F*_1,45_ = 6.50, *p* = .014) spent in the open arms of an elevated plus maze decrease upon refeeding in male and female rats. **(G)** The total distance moved in an elevated plus maze is not significantly changed by the feeding status (ANOVA, *F*_1,45_ = 2.89, *p* = .10). **(H)** Average heatmaps of fasted or refed rats during a 5-minute elevated plus maze test. **(I)** Startle amplitude in response to acoustic stimuli is increased in refed compared with fasted rats (ANOVA 90 dB: *F*_1,45_ = 0.26, *p* = .61; 105 dB: *F*_1,45_ = 7.54, *p* = .009; 120 dB *F*_1,45_ = 4.61, *p* = .037). **(J)** Time to start eating a novel food increases in refed compared with fasted rats (ANOVA, *F*_1,91_ = 34.31, *p* < .0001). *p < .05; **p < .01; ***p < .001. ANOVA, analysis of variance; NS, not significant.

The effect of the feeding status on anxiety-like behavior was assessed in overnight fasted or 1-hour ad libitum refed rats using 4 anxiety paradigms. In an open field test (Figure 1A-D), refed rats covered less distance and entered the center zone less frequently (Figure 1A, B) than fasted rats. No significant changes in the total distance traveled were detected (Figure 1C). In the elevated plus maze (Figure 1E, F), refed rats decreased the distance (Figure 1E) and time (Figure 1F) spent in open arms, but not the total distance (Figure 1G), compared with fasted rats. As locomotor activity tended to be reduced in refed rats, we verified the robustness of these results by adding total locomotor activity as covariate and found similar results (Figure S1A-D). Refed rats showed increased acoustic startle reflex compared with fasted rats in a locomotor activity–independent test (Figure 1I). Finally, refed rats showed a greater latency to start eating a novel food than fasted rats (Figure 1J). These effects are likely independent of changes in hunger, as they remain significant after statistical adjustment for 30-minute post-test food intake (Figure S1E, F). Altogether, these results indicate that 1-hour ad libitum refeeding increases anxiety-like behavior in male and female rats (Figure S6A), independent of changes in locomotor activity or hunger.

### Selective Activation of Vagal Afferents Projecting to the Gastrointestinal Tract Increases Anxiety-like Behavior

**Figure 2.**
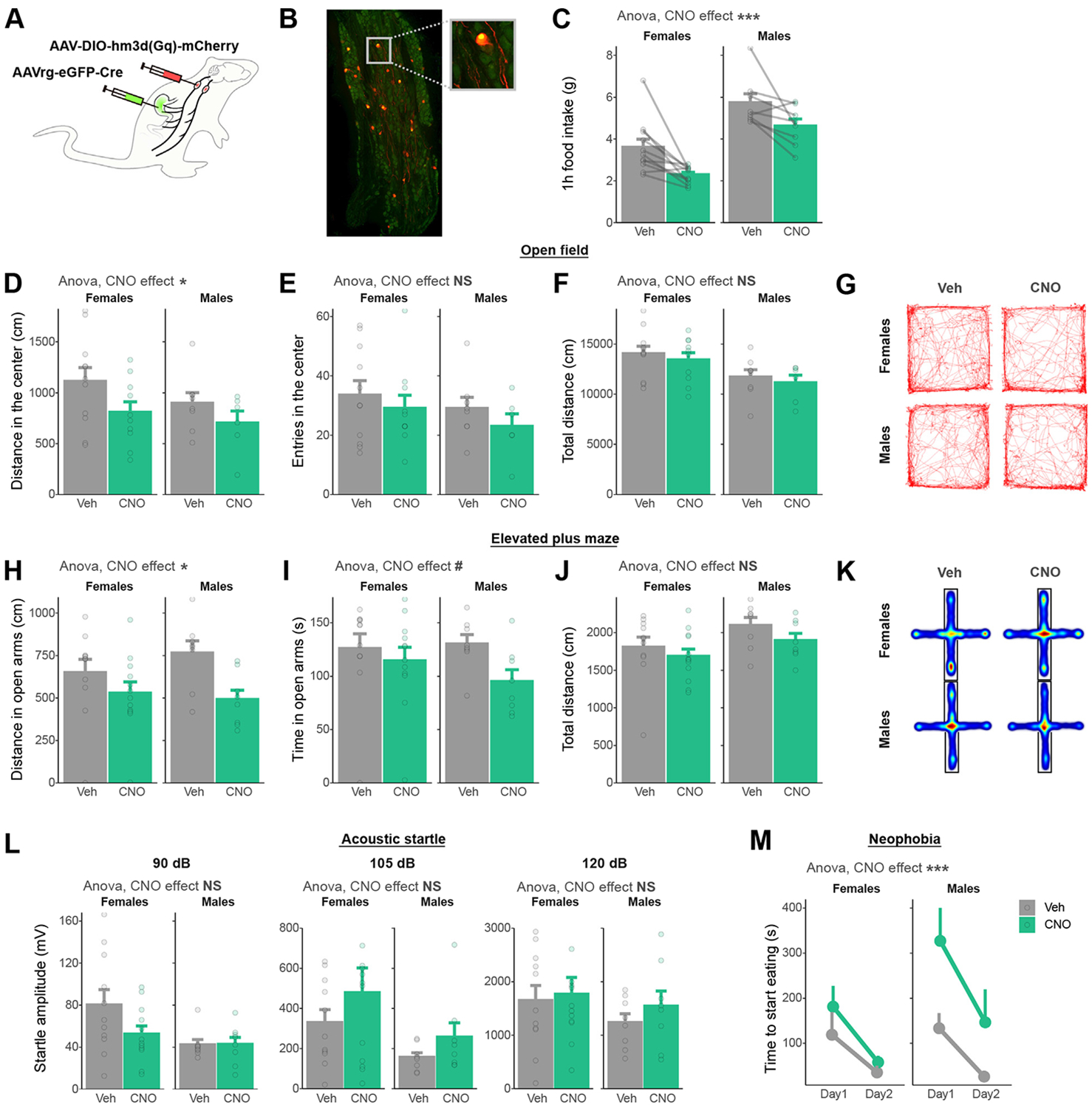
Chemogenetic activation of vagal afferents projecting to the gastrointestinal tract increases anxiety-like behavior in male and female rats. **(A)** Dual viral injection strategy used to selectively activate vagal afferents terminating in the stomach and duodenum. **(B)** Representative picture of viral vector expression in the nodose ganglia [green: eGFP from retrograde AAV-Cre; red: mCherry from AAV-DIO-hm3D(Gq)]. **(C)** One-hour food intake decreases after CNO injection compared with Veh (ANOVA, *F*_1,34_ = 14.55, *p* = 5.48 ×10^−4^). **(D)** Distance (ANOVA, *F*_1,32_ = 4.73, *p* = .037) but not **(E)** number of entries (ANOVA, *F*_1,32_ = 1.25, *p* = .271) in the center of an open field decreases after CNO administration in male and female rats. **(F)** The total distance moved in an open field is not significantly changed by CNO injection (ANOVA, *F*_1,32_ = 0.67, *p* = .42). **(G)** Representative traces of Veh- or CNO-injected rats during a 30-minute open field test. **(H)** Distance (ANOVA, *F*_1,36_ = 6.57, *p* = .015) spent in the open arms of an elevated plus maze decreases after CNO injection compared with vehicle. **(I)** Time (ANOVA, *F*_1,36_ = 2.93, *p* = .096) spent in the open arms of an elevated plus maze is not significantly decreased by CNO injection. **(J)** The total distance moved in an elevated plus maze is not significantly changed by CNO injection (ANOVA, *F*_1,36_ = 1.96, *p* = .17). **(K)** Average heatmaps of Veh- or CNO-injected rats during a 5-minute elevated plus maze test. **(L)** Startle amplitude in response to acoustic stimuli is not significantly changed by CNO injection (ANOVA 90 dB: *F*_1,36_ = 2.55, *p* = .12; 105 dB: *F*_1,36_ = 2.09, *p* = .16; 120 dB *F*_1,36_ = 0.11, *p* = .74). **(M)** Time to start eating a novel food increases in CNO-injected compared with fasted rats (ANOVA, *F*_1,73_ = 15.40, *p* = .0002). #*p* < .1; *p < .05; ***p < .001. ANOVA, analysis of variance; CNO, clozapine *N*-oxide; eGFP, enhanced green fluorescent protein; NS, not significant; Veh, vehicle.

One-hour ad libitum refeeding substantially increased stomach contents compared with an overnight fast (Figure S1G, L) but only mildly increased the intestinal contents (Figure S1H-L). Considering that vagal afferents are activated by stretch and nutrient-derived signals from the gastrointestinal tract [see (20) for review], we sought to test whether the activation of these neurons increases anxiety-like behavior. We adapted a dual injection strategy previously published in mice (14) to selectively activate vagal afferents projecting to the stomach and the duodenum with chemogenetics in rats. Briefly, the stomach and duodenum of rats were injected with a retrogradely transported, Cre-expressing AAV (adeno-associated virus). A second construct injected into the nodose ganglia allowed the Cre-dependent expression of the designer receptor hm3D(Gq) and mCherry specifically into vagal afferents projecting to the stomach and the duodenum (Figure 2A). In addition, a control cohort was generated to selectively express mCherry but not hm3D(Gq) in the gut-innervating vagal afferents (Figure S4A, B). Overlapping expression of the retrograde AAV-induced eGFP (enhanced green fluorescent protein) and Cre-dependent mCherry was visualized in the nodose ganglia (Figure 2B). Consistent with the known role of vagal afferent neurons in satiation [see (21) for review], clozapine *N*-oxide (CNO, 2 mg/kg intraperitoneally in all tests) injections reduced 1-hour food intake compared with vehicle injections in hm3d(Gq)-expressing rats (Figure 2C) but not in non–hm3d(Gq)-expressing control rats (Figure S4C).

Chemogenetic activation of gut-innervating vagal afferents reduced the distance traveled in the center zone of the open field (Figure 2D) but not the number of entries into the center zone (Figure 2E) or the total distance covered in the open field (Figure 2F). In the elevated plus maze, chemogenetic activation of gut-innervating vagal afferents reduced the distance (Figure 2H) spent in the open arms without significantly affecting locomotor activity (Figure 2J). CNO injection also tended to reduce the time spent in open arms (Figure 2I). The modulation of anxiety-like behavior by CNO was not dependent on subthreshold changes in locomotor activity (Figure S2A, D). The startle amplitude to acoustic stimuli, however, was not modulated by the activation of vagal afferents (Figure 2L). Finally, rats injected with CNO showed increased latency to start eating a novel food (Figure 2M), even after statistical adjustment for changes in hunger (Figure S2E, F). Importantly, CNO injections in non-DREADD (designer receptor exclusively activated by designer drugs) expressing control rats did not modulate any surrogates of anxiety-like behavior (Figure S4D-M). Overall, these results indicate that the selective activation of vagal afferent neurons projecting to the stomach and duodenum induces anxiety-like behavior in rats (Figure S6B).

### Selective Inhibition of Gut-Innervating Vagal Afferent Neurons Attenuates the Anxiogenic Effects of Refeeding

**Figure 3.**
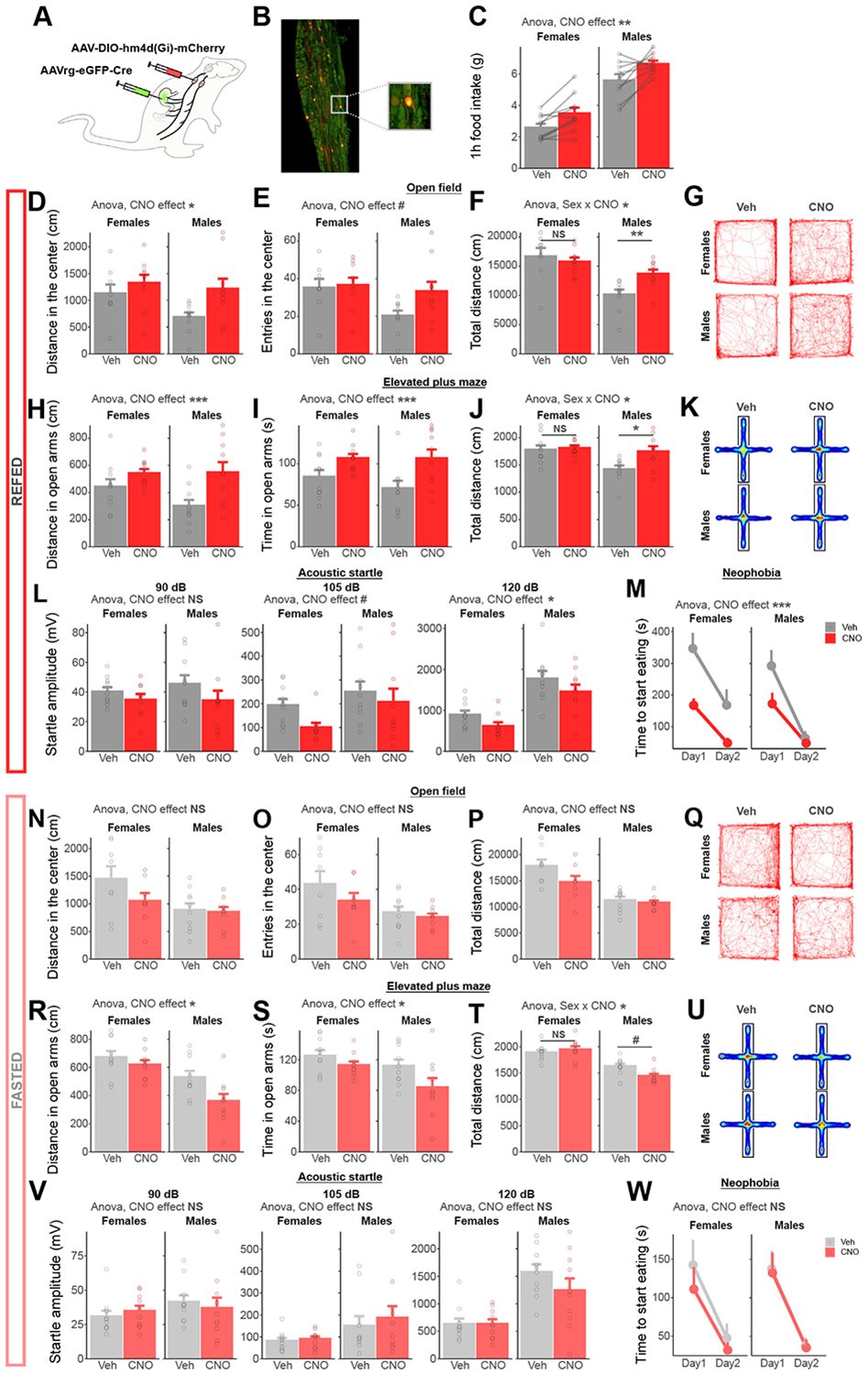
Chemogenetic inhibition of vagal afferents projecting to the gastrointestinal tract blocks the anxiogenic effect of refeeding. **(A)** Dual viral injection strategy used to selectively inhibit vagal afferents terminating in the stomach and duodenum. **(B)** Representative picture of viral vector expression in the nodose ganglia [green: eGFP from retrograde AAV-Cre; red: mCherry from AAV-DIO-hm4D(Gi)]. **(C)** One-hour food intake increases after CNO injection compared with Veh (ANOVA, *F*_1,35_ = 9.49, *p* = .004). **(D)** Distance in the center of an open field increases after CNO administration in refed rats (ANOVA, *F*_1,35_ = 5.94, *p* = .020). **(E)** The number of entries in the center of an open field shows a trend toward increase after CNO administration in refed rats (ANOVA, *F*_1,35_ = 3.19, *p* = .083). **(F)** The effects of CNO on the total distance moved in an open field test depend on sex in refed rats (ANOVA sex × CNO interaction, *F*_1,34_ = 5.72, *p* = .022, Holm-adjusted comparisons, females *p* = .57, males *p* = .0012). **(G)** Representative traces of Veh- or CNO-injected rats (refed) during a 30-minute open field test. **(H)** CNO increases the distance traveled in the open arms of an elevated plus maze in refed rats (ANOVA, *F*_1,37_ = 10.09, *p* = .003). **(I)** CNO increases the time spent in the open arms of an elevated plus maze in refed rats (ANOVA, *F*_1,37_ = 11.97, *p* = .001). **(J)** The effects of CNO on the total distance moved in an elevated plus maze depend on sex in refed rats (ANOVA sex × CNO interaction, *F*_1,37_ = 4.88, *p* = .047, Holm-adjusted comparisons, females *p* = .71, males *p* = .012). **(K)** Average heatmaps of Veh- or CNO-injected rats (refed) during a 5-minute elevated plus maze test. **(L)** Startle amplitude in response to acoustic stimuli is increased after CNO injection in refed rats (ANOVA 90 dB: *F*_1,36_ = 2.68, *p* = .11; 105 dB: *F*_1,36_ = 2.82, *p* = .10; 120 dB *F*_1,36_ = 4.14, *p* = .049). **(M)** CNO inhibition decreases the time to start eating a novel food (ANOVA, *F*_1,72_ = 18.76, *p* = .00047). **(N)** Distance in the center of an open field is not modulated by CNO administration in fasted rats (ANOVA, *F*_1,32_ = 1.78, *p* = .19). **(O)** The number of entries in the center is not modulated by CNO administration in fasted rats (ANOVA, *F*_1,32_ = 1.71, *p* = .20). **(P)** The total distance moved in an open field test is not modulated by CNO administration in fasted rats (ANOVA, *F*_1,32_ = 2.12, *p* = .16). **(Q)** Representative traces of Veh- or CNO-injected rats (fasted) during a 30-minute open field test. **(R)** CNO decreases the distance traveled in the open arms of an elevated plus maze in fasted rats (ANOVA, *F*_1,36_ = 6.52, *p* = .015). **(S)** CNO decreases the time spent in the open arms of an elevated plus maze in fasted rats (ANOVA, *F*_1,37_ = 5.67, *p* = .023). **(T)** The effects of CNO on the total distance moved in an elevated plus maze depend on sex in refed rats (ANOVA sex × CNO interaction, *F*_1,36_ = 5.71, *p* = .022, Holm-adjusted comparisons, females *p* = .48, males *p* = .010). **(U)** Average heatmaps of Veh- or CNO-injected rats (fasted) during a 5-minute elevated plus maze test. **(V)** Startle amplitude in response to acoustic stimuli is not modulated by CNO injection in fasted rats (ANOVA 90 dB: *F*_1,37_ = 0.001, *p* = .97; 105 dB: *F*_1,37_ = 0.40, *p* = .53; 120 dB *F*_1,37_ = 1.28, *p* = .27). **(W)** CNO inhibition does not modulate the time to start eating a novel food in fasted rats (ANOVA, *F*_1,76_ = 0.83, *p* = .37). #*p* < .1; *p < .05; **p < .01; ***p < .001. ANOVA, analysis of variance; CNO, clozapine *N*-oxide; eGFP, enhanced green fluorescent protein; NS, not significant; Veh, vehicle.

As both feeding and the selective activation of gut-innervating vagal afferents increased anxiety-like behavior in male and female rats, we subsequently tested whether gut-innervating vagal afferents are necessary for the anxiogenic effects of refeeding. We used a dual injection strategy to selectively express the inhibitory DREADD receptor hm4D(Gi) into vagal afferent neurons projecting to the stomach and duodenum (Figure 3A). Overlapping expression of the retrograde eGFP and mCherry was visualized in the nodose ganglia (Figure 3B). In addition, CNO injection increased 1-hour food intake in hm4D(Gi)-expressing rats (Figure 3C) but not in their corresponding control rats (Figure S4C).

In the open field test, chemogenetic inhibition of gut-innervating vagal afferents increased the distance traveled and tended to increase the number of entries in the center zone in refed rats (Figure 3D-G) but not in fasted rats (Figure 3N-Q). In the elevated plus maze test, chemogenetic inhibition of gut-innervating vagal afferents increased the distance traveled and time spent in the open arms in refed rats (Figure 3H-K) but not in fasted rats (Figure 3R-U). In both tests, total locomotor activity was modulated in a sex-dependent manner (Figure 3F, J). After adding locomotor activity as a covariate, the anxiolytic effects of CNO in refed rats remained significant in the elevated plus maze test but not the open field (Figure S3A-D). In a locomotor-independent test, chemogenetic inhibition of gut-innervating vagal afferents decreased the startle responses to acoustic stimuli in refed rats (Figure 3L) but not in fasted rats (Figure 3V). Similarly, chemogenetic inhibition of gut-innervating vagal afferents blocked the feeding-induced increase in latency to eat a novel food in refed rats (Figure 3M) but not in fasted rats (Figure 3W), an effect not mediated by associated changes in hunger (Figure S3E-G). Altogether, these results demonstrate that the increased anxiety-like behavior in refed rats requires vagal afferent gut-brain signaling (Figure S6C).

### Chronic Lesion of Gastrointestinal Vagal Afferents Decreases Anxiety-like Behavior in a Sex-Dependent Manner

**Figure 4.**
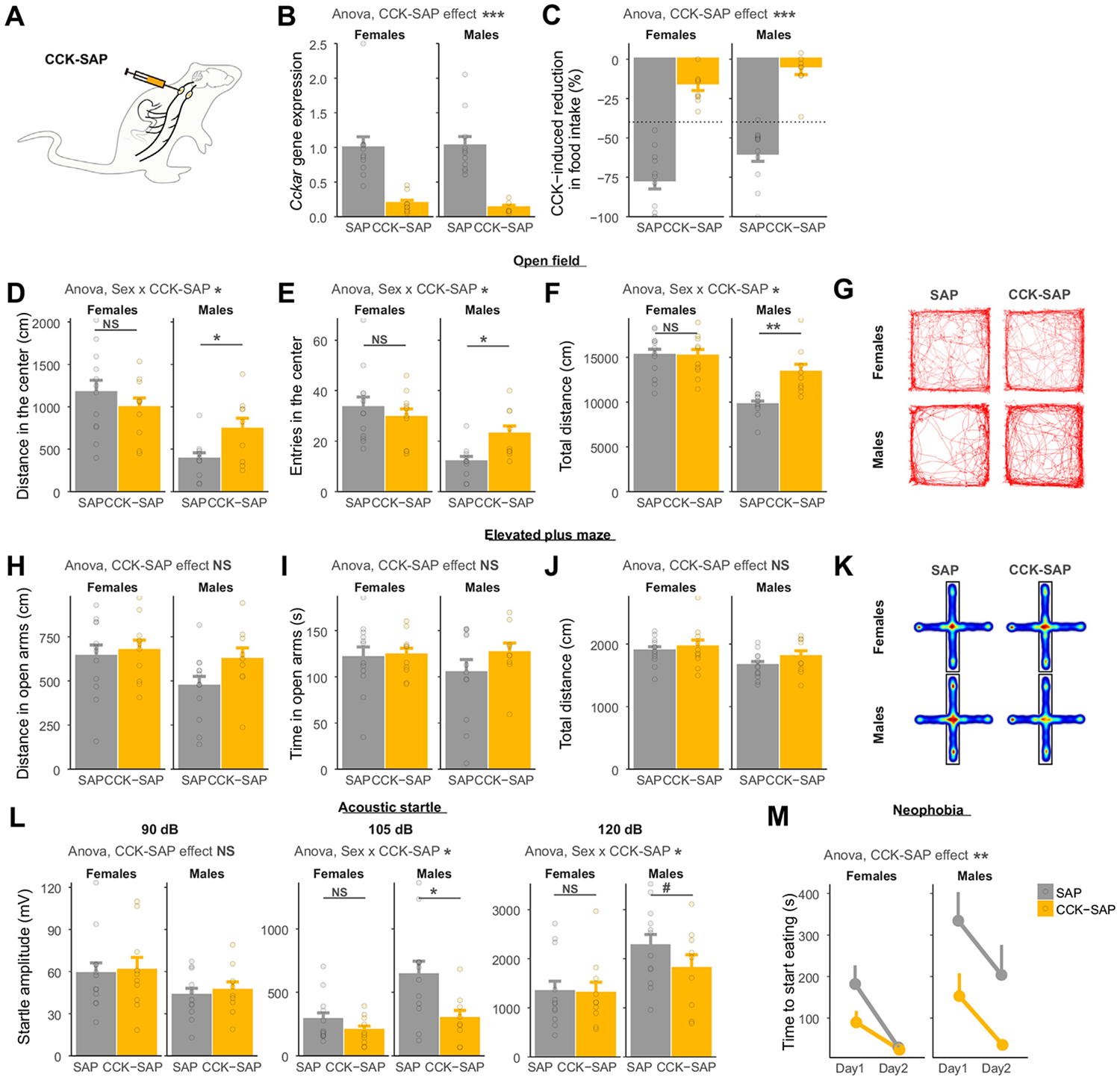
Chronic lesion of gastrointestinal vagal afferents decreases anxiety-like behavior in a sex-dependent manner. **(A)** Injection strategy used to selectively lesion gastrointestinal vagal afferents. **(B)** CCK-SAP reduces the nodose ganglia expression of *Cckar* compared with SAP (ANOVA, *F*_1,34_ = 42.17, *p* = 1.98 × 10^−7^). **(C)** The anorexigenic effect of intraperitoneal CCK is abolished in CCK-SAP rats (ANOVA, *F*_1,39_ = 119.14, *p* = 2.0 × 10^−13^). **(D)** The effect of CCK-SAP on the distance in the center of an open field depends on sex (ANOVA, sex × group interaction, *F*_1,37_ = 4.63, *p* = .038). CCK-SAP increases the distance spent in the center of an open field in males (Holm-adjusted comparisons *p* = .028) but not in females (*p* = .36). **(E)** The effect of CCK-SAP on the number of entries in the center of an open field depends on sex (ANOVA, sex × group interaction, *F*_1,37_ = 4.52, *p* = .040). CCK-SAP increases the number of entries in the center of an open field in males (Holm-adjusted comparisons *p* = .010) but not in females (*p* = .50). **(F)** The effect of CCK-SAP on total distance moved in an open field depends on sex (ANOVA, sex × group interaction, *F*_1,37_ = 6.99, *p* = .012). CCK-SAP increases the total distance moved in males (Holm-adjusted comparisons *p* = .0013) but not in females (*p* = .94). **(G)** Representative traces of SAP and CCK-SAP rats during a 30-minute open field test. **(H)** Distance (ANOVA, *F*_1,40_ = 2.20, *p* = .15) and **(I)** time (ANOVA, *F*_1,40_ = 1.03, *p* = .32) spent in the open arms of an elevated plus maze do not differ between SAP and CCK-SAP rats. **(J)** The total distance moved in an elevated plus maze is not significantly affected by CCK-SAP (ANOVA, *F*_1,40_ = 1.50, *p* = .23). **(K)** Average heatmaps of SAP and CCK-SAP rats during a 5-minute elevated plus maze test. **(L)** Startle amplitude in response to acoustic stimuli is modulated by CCK-SAP in a sex-dependent manner (ANOVA 90 dB: group effect *F*_1,38_ = 0.17, *p* = .69; 105 dB: sex × group interaction *F*_1,39_ = 8.21, *p* = .007; Holm-adjusted comparisons males *p* = .021, females *p* = .21; 120 dB: sex × group interaction *F*_1,39_ = 9.12, *p* = .004; Holm-adjusted comparisons males *p* = .10, females *p* = .91). **(M)** CCK-SAP decreases the time to start eating a novel food compared with SAP (ANOVA, *F*_1,81_ = 10.6, *p* = .002). #*p* < .1; *p < .05; **p < .01; ***p < .001. ANOVA, analysis of variance; CCK, cholecystokinin; CeA, central nucleus of the amygdala; HSV, herpes simplex virus; NS, not significant; SAP, saporin.

We next asked whether a chronic interruption of vagal feedback from the gastrointestinal tract to the brain leads to sustained changes in anxiety-like behavior, independent of acute food intake. To achieve this, we used a previously validated method based on the bilateral injection into the nodose ganglia of CCK-SAP (Figure 4A) (22). In our cohort, CCK-SAP injections decreased *Cckar* expression in the nodose ganglia by 83% in males and 89% in females (Figure 4B) and strongly attenuated the anorexigenic response to intraperitoneal cholecystokinin injections (Figure 4C). As previously reported (23), chow-fed CCK-SAP rats showed no changes in body weight or 24-hour food intake (Figure S5A, B).

In an open field test, a lesion of gastrointestinal vagal afferents affected anxiety-like behavior in a sex-dependent manner. Male CCK-SAP rats but not females traveled less distance and entered less into the center zone than control rats (Figure 4D-G). In the elevated plus maze test, there was a consistent trend for increased distance and time in the open arms in male CCK-SAP rats but not in females (Figure 4H-K). In both paradigms, however, changes in locomotor activity mediate the effect of CCK-SAP on anxiety surrogate variables (Figure S5C-F). In a locomotor activity–independent test, however, CCK-SAP decreased the amplitude of startle responses in male rats but not in female rats (Figure 4L). Finally, CCK-SAP decreased the latency to start eating a novel food in both sexes (Figure 4M; Figure S5G, H), although males showed a longer-lasting effect. Taken together, these results indicate that the permanent absence of vagal afferent feedback from the gastrointestinal tract decreases anxiety-like behavior in a sex-dependent manner (Figure S6D).

### Vagal Afferent Neurons Are Polysynaptically Connected to the CeA

**Figure 5.**
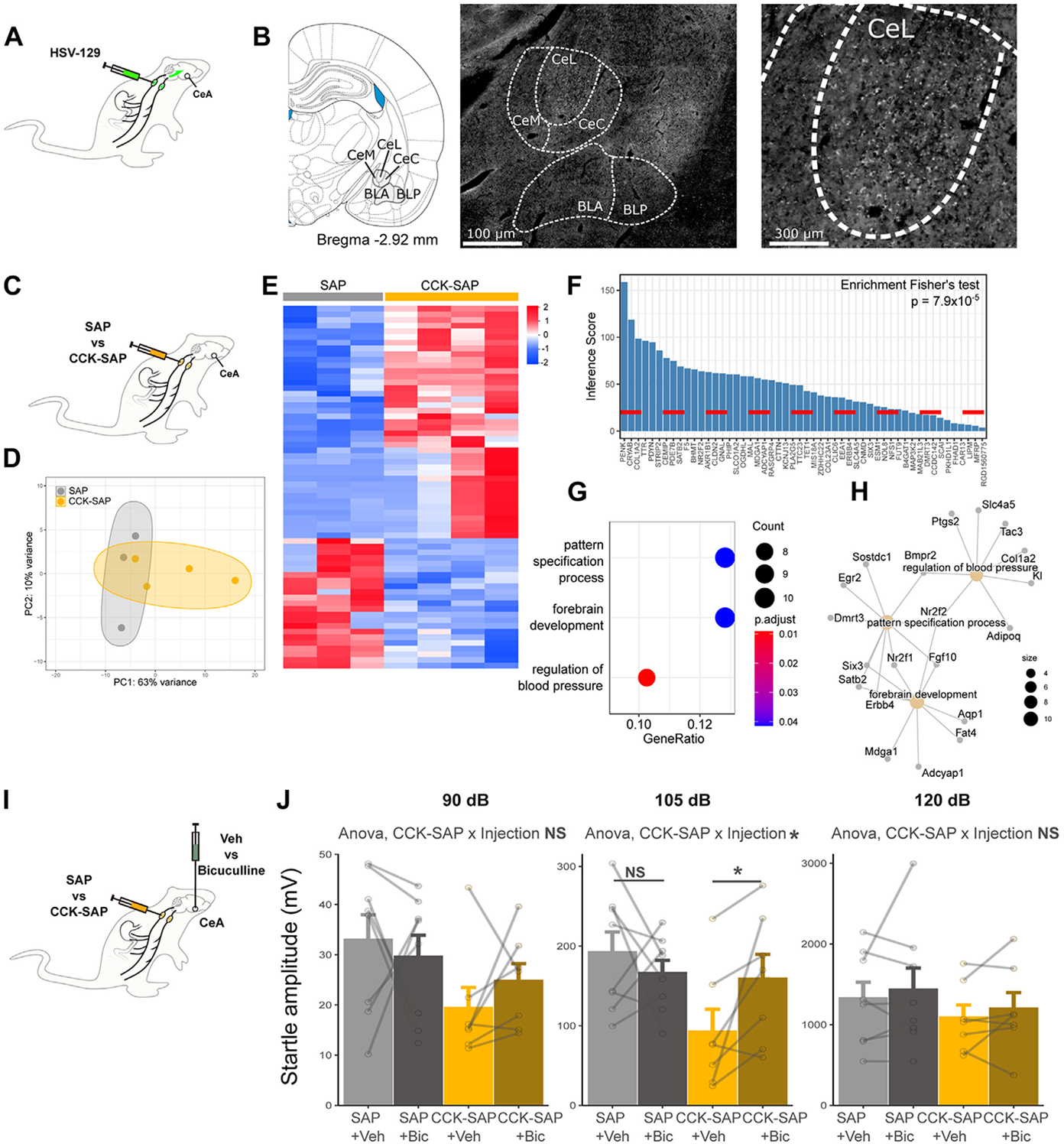
GABAergic signaling in the central amygdala is modulated by vagal afferents and underlies the anxiety-reducing effects of CCK-SAP in male rats. **(A)** Strategy used for anterograde polysynaptic tracing from the nodose ganglia with HSV-129. **(B)** Representative pictures of HSV-129 labeling in the amygdala 5 days after HSV-129 injection into the nodose ganglia (CeL, CeM, CeC, BLA, BLP). **(C)** Strategy used for RNA sequencing of CeA micropunches after CCK-SAP or SAP in male rats. **(D)** PC analysis plot of CeA gene expression profiles in CCK-SAP and control rats. **(E)** Heatmaps of gene expression (*z* score) for significant DEGs in the CeA of CCK-SAP compared with control rats (adjusted *p* < .05). **(F)** Inference scores of DEGs in the CeA associated with anxiety disorders according to the Comparative Toxicogenomics Database. **(G)** Significantly enriched biological processes among DEGs in the CeA (adjusted *p* < .05). **(H)** DEGs associated with the 3 significantly enriched biological processes in the CeA. **(I)** Strategy used for the bilateral administration of the GABA_A_ receptor antagonist bicuculline in CCK-SAP or SAP male rats. **(J)** Bicuculline injection into the CeA increases startle amplitude in response to a 105-dB acoustic stimulus in CCK-SAP male rats but not in SAP male rats (within-subject ANOVA CCK-SAP × injection interaction, *F*_1,13_ = 4.90, *p* = .045; Holm-adjusted comparison in SAP rats *p* = .42, in CCK-SAP rats *p* = .041). Bicuculline does not significantly modulate startle amplitude in response to 90 dB (within-subject ANOVA; injection effect *F*_1,13_ = 0.050, *p* = .83; CCK-SAP × injection, *F*_1,13_ = 0.947, *p* = .35) and 120 dB (within-subject ANOVA; injection effect *F*_1,13_ = 1.050, *p* = .32; CCK-SAP × injection, *F*_1,13_ = 0.0001, *p* = .99) stimuli. *p < .05. ANOVA, analysis of variance; Bic, bicuculline; BLA, basolateral amygdaloid nucleus, anterior part; BLP, basolateral amygdaloid nucleus, posterior part; CCK, cholecystokinin; CeA, central nucleus of the amygdala; CeC, central amygdaloid nucleus, capsular part; CeL, central amygdaloid nucleus, lateral division; CeM, central amygdaloid nucleus, medial division; DEGs, differentially expressed genes; GABA, gamma-aminobutyric acid; NS, not significant; PC, principal component; SAP, saporin; Veh, vehicle.

The expression of anxiety-like behavior depends on the output of distributed neural circuits that include the CeA and the BNST. We therefore tested whether a synaptic circuit exists between vagal afferent neurons and the CeA or BNST. For this, the anterograde polysynaptic viral tracer herpes simplex virus (HSV)-129 was injected bilaterally into the nodose ganglia, and the spread of infection was visualized 5 days after injection (Figure 5A). As previously reported in mice (14), HSV labeling was visible in the nucleus tractus solitarii where vagal afferent neuron terminals are found (Figure S7A). In addition, HSV labeling was found in the CeA, especially in the lateral division (central amygdaloid nucleus, lateral division) (Figure 5B). Limited labeling, however, was detected within the BNST (Figure S7B). Overall, these data indicate that a polysynaptic neuronal circuit links vagal afferent neurons to the CeA in rats.

### Vagal Deafferentation of the Gastrointestinal Tract Selectively Modulates the Expression of Genes Associated With Anxiety and GABAergic Synapses in the CeA of Male Rats

We next hypothesized that gut-innervating vagal afferents modulate anxiety-associated pathways in the CeA but not in the BNST. To test this, brain micropunches of the CeA and BNST of male CCK-SAP and SAP control rats were submitted to next-generation messenger RNA sequencing (Figure 5C). Overall, the gene expression profiles of both brain regions partially differed between CCK-SAP and SAP control rats (Figure 5D; Figure S7C). Differential expression analysis revealed 64 differentially expressed genes in the CeA (41 increased vs. 23 decreased in CCK-SAP compared with SAP control rats; adjusted *p* < .05) (Figure 5F; Table S1), whereas 15 differentially expressed genes were found in the BNST (9 increased vs. 6 decreased) (Figure 5K; Table S2).

To investigate whether gastrointestinal vagal deafferentation selectively affects anxiety-associated pathways, we used the inference score for Anxiety Disorders given by the Comparative Toxicogenomics Database (24) (Figure 5G, L). Gastrointestinal vagal deafferentation selectively modulated genes associated with anxiety disorders in the CeA (*p* = 7.9 × 10^−5^) (Figure 5F) but not in the BNST (not significant) (Figure S7E) of male rats. Notably, gastrointestinal vagal deafferentation affected genes previously associated with anxiety modulation in the mouse amygdala (*Cldn2*, *Mfrp*, *Ttr*, *F5*, *Clic6*) (see Table S1), although in opposite direction compared with acute or early-life stressors (25,26). We then determined the enriched biological processes in the CeA of male rats using Gene Ontology terms (Figure 5G). Pattern specification and forebrain development were the 2 highest-ranked biological processes (Figure 5H), suggesting that gastrointestinal vagal deafferentation broadly reorganizes neuronal transmission in the amygdala. Specifically, genes within these processes are known modulators of GABAergic (gamma-aminobutyric acidergic) synapses, such as *Adcyap1* (27), *Mdga1* (28), *Erbb4* (29), or *Nr2f1* (30) (see Table S1). The expression of these genes was increased in CCK-SAP rats compared with control rats, strongly suggesting that gastrointestinal vagal deafferentation enhances inhibitory synaptic transmission in the CeA of male rats. In female CCK-SAP rats, which showed no changes in anxiety-like behavior compared with control rats (Figure 4), the extent of gene modulation in the CeA was more limited than in males (13 vs. 64 differentially expressed genes) (Figure S7F, G and Table S2) but still biased toward genes known to affect anxiety (*p* = .021) (Figure S7H). In females, CCK-SAP did not significantly increase the expression of the previously identified GABA-related genes (Figure S7I-N), although most genes showed the same trend as in the male cohort.

Altogether, these findings associate the anxiolytic effects of CCK-SAP seen in male rats with a potentiation of GABAergic signaling in the amygdala.

### The Anxiolytic Effects of Gastrointestinal Vagal Deafferentation Necessitate GABAergic Signaling in the CeA

We then tested whether the anxiolytic effects of vagal deafferentation seen in male rats require intact GABAergic signaling in the CeA. For this, we infused the GABA_A_ receptor antagonist bicuculline bilaterally (100 pmol in 0.2 μL per side) into the CeA of male CCK-SAP rats or their corresponding control rats (Figure 5I) and tested their anxiety-like behavior in the acoustic startle test. Bicuculline increased the startle amplitude of CCK-SAP rats but not control rats in response to a 105 dB stimulus (Figure 5J). A similar trend was also observed in response to a 90 dB stimulus but not to a 120 dB. Altogether, this pharmacological approach supports the idea that the anxiety-like effects of gastrointestinal vagal afferents are mediated by GABAergic signaling in the CeA of male rats.

## DISCUSSION

The interoceptive modulation of anxiety can be observed in rodents following changes in metabolic states sensed by the gastrointestinal tract (6-11). The identity of the gut-brain pathway mediating this association remained unknown. Here, we first expanded prior literature indicating that fasting and refeeding bidirectionally modulate anxiety-like behavior in rats (6-11) by demonstrating similar outcomes in female rats. Using selective chemogenetic activation of gut-innervating vagal afferents, we showed that increasing the activity of these neurons is anxiogenic. Conversely, chemogenetic inhibition of gut-innervating vagal afferents blocked the increase in anxiety-like behavior induced by feeding. In addition, we found that a chronic interruption of vagal feedback from the gastrointestinal tract to the brain reduces anxiety-like behavior in male rats but not in female rats. Finally, the reduced anxiety-like behavior seen in male rats after gastrointestinal vagal deafferentation is associated with a modulation of the CeA transcriptome indicative of a potentiation of inhibitory synaptic transmission. Blocking GABAergic signaling in the CeA abolishes the anxiolytic effects of gastrointestinal vagal deafferentation. Altogether, our findings demonstrate that gut-innervating vagal afferents provide the necessary gut-brain feedback for the metabolic tuning of anxiety and highlight sex differences in the susceptibility to impairments of this gut-brain feedback.

### Novel Selective Approaches for the Study of Vagal Afferents Unlock Investigation of Their Role in Behavior

We hypothesized that vagal afferent neurons, specifically those terminating into the gastrointestinal tract, mediate the metabolic tuning of anxiety-like behavior in rats. Despite a longstanding history of method development for vagal research, classic approaches (20) such as vagotomy or SDA also affect vagal efferents, as well as afferents terminating outside the gastrointestinal tract. Thus, while a previous study indicated that SDA reduced innate anxiety-like behavior in rats (16), it remained unclear whether gut-projecting vagal afferents mediated this anxiolytic effect (31). Here, we used a previously validated toxin-based approach that is specific to gastrointestinal vagal afferents and spares vagal efferents (22), thus complementing previous findings in the SDA model (16).

Recently, AAV administered into the nodose ganglia of transgenic mice allowed to interrogate the function of genetically defined vagal afferent subtypes (12,32-34). This technique is, however, not specific to anatomical projections, as genetically defined subsets may project to multiple organs, and conversely, a single organ receives projections form multiple genetically defined subsets (12,35). Given these limitations, we adapted a novel 2-vector strategy recently documented in mice (14) to selectively and reversibly activate or inhibit vagal afferent neurons projecting to the gastrointestinal tract with chemogenetics. This allowed for the investigation of their role in anxiety-like behavior with a time resolution compatible with fasting-feeding cycles.

Like previous studies, however, we did not investigate the gastrointestinal signals that trigger the vagal modulation of anxiety. Gut-projecting vagal afferents mediate both mechanical and nutrient-derived signals to the brain, and future studies should determine which of these signals trigger the vagal-to-amygdala circuit.

### The Effects of Eating on Anxiety: Relationship With Hunger and Locomotion

Our study aimed to measure anxiety-like behavior in the context of food intake or in response to the modulation of neurons known to be involved in the control of food intake. Thus, a challenge we faced was the experimental dissociation of anxiety-like behavior from behavioral correlates of hunger and satiation. The well-described “behavioral satiety sequence” is a stereotypical series of behavioral changes including changes in locomotion that follow eating and satiation in rodents (36,37). Yet, most commonly used rodent anxiety tests, namely, the open field and elevated plus maze, depend on locomotion to evaluate the animals’ conflict between their drive to explore novel environments and their avoidance of open spaces. We first addressed this challenge by statistically adjusting a posteriori for changes in locomotor activity, using total distance moved as a covariate in analyses of covariance of the open field and elevated plus maze results. In line with previous findings in SDA rats, feeding or vagal manipulations modulate markers of anxiety-like behavior in the open field and elevated plus maze independent of changes in total locomotor activity (16). In addition, the food neophobia test is based on the intake of a novel food and is therefore influenced by changes in appetite. Thus, we statistically adjusted for changes in appetite using the 30-minute post-test food intake as a covariate in analyses of covariance. After these statistical adjustments, we still consistently found that the activity of gut-innervating vagal afferents modulates markers of anxiety-like behavior. In addition, to eliminate all confounding by locomotion or hunger a priori, we used the acoustic startle test, which assesses anxiety-like behavior independently of locomotion or food intake, and found a modulation of anxiety markers in all but one cohort. Thus, we conclude that the activity of gut-innervating vagal afferents modulates hunger but that hunger does not lie in the causal pathway between vagal activity and changes in anxiety-like behavior. In other words, postprandial changes in hunger and anxiety-like behavior are physiologically concomitant but mechanistically distinguishable.

### Central Anxiety Pathways Under Vagal Influences

The expression of anxiety-like behavior depends on the output of distributed neural circuits that include mainly the BNST and CeA. We reasoned that meal-related vagal signals would primarily affect the CeA to elicit anxiety-like responses time-locked to changes in the feeding status, rather than affecting the BNST, classically associated with long-term anxiety changes (38). Moreover, the CeA integrates meal-related interoceptive cues, e.g., the glucagon-like peptide-1 (39) and ghrelin (11), to tune anxiety-like behavior.

Our study identifies the CeA as a brain area receiving synaptic inputs from vagal afferents, consistent with previous observations in mice (14) and retrograde tracing from the gastrointestinal tract (40). Although our experiments did not aim at defining the exact circuit linking gastrointestinal vagal afferents to the CeA, earlier findings in mice point toward a nucleus tractus solitarii-to-CeA pathway involving a relay via the parabrachial nucleus (14).

In contrast to the CeA, we observed limited HSV labeling in the BNST. It is possible, however, that the vagus nerve and the BNST are distantly connected and that HSV would require extended incubation before extensive labeling could be visualized in the BNST. In mice, HSV labeling is visible after 5 days in the BNST but not at earlier time points (14). However, in line with the limited labeling, we found no anxiety-relevant transcriptome changes in the BNST following gastrointestinal vagal deafferentation.

In the CeA, however, we found that a chronic disruption of gut-brain vagal feedback selectively modulates anxiety-related genes in male rats. For example, *Cldn2*, *Mfrp*, *Ttr*, *F5*, or *Clic6* are also modulated in the mice amygdala after anxiogenic exposure to early-life stressors (though in opposite directions compared with CCK-SAP) (25,26).

Information processing within the amygdala is dependent on inhibitory control (41): abnormalities in inhibitory transmission within the amygdala play a key role in anxiety disorders [see (41) for review]. Conversely, benzodiazepines, a class of GABA_A_ receptor agonists, are widely used for the treatment of anxiety disorders (42). Previous evidence for the effects of vagal inputs on GABAergic neurotransmission in the CNS was found in vagotomized mice (17) and SDA rats (16), although not specifically in the CeA. Here, we found that gastrointestinal vagal deafferentation was associated with the increased expression of genes regulating GABAergic synapses in the CeA. Notably, CCK-SAP increases *Adcyap1* expression, which is the precursor gene for the pituitary adenylate cyclase-activating polypeptide, a known potentiator of GABA release in the CeA of rats (27). Likewise, the expression of *Erbb4* is increased in CCK-SAP rats compared with control rats: mice with high anxiety phenotype display low levels of ErbB4 in the amygdala, and administration of its ligand neuregulin 1 is anxiolytic while enhancing GABAergic neurotransmission (29). Similarly, multiple genes increased in the amygdala of CCK-SAP rats, such as *Nr2f1* (30), *Nr2f2* (43), *Mdga1* (28), or *Satb2* (44), are primarily characterized for their role in regulating and maintaining inhibitory synapses in the developing brain. Overall, our results indicate that the decreased anxiety-like behavior induced by gastrointestinal vagal deafferentation associates with increased inhibitory control in the CeA. Indeed, in male rats, blocking GABAergic signaling in the CeA abolishes the anxiolytic effects of gastrointestinal vagal deafferentation.

### Chronic Disruption of Vagal afferent Neurons Affects Anxiety-like Behavior in a Sex-Specific Manner

Nearly all previous studies investigating the role of vagal afferents on behavioral outcomes (14-16,45-47) included only male rodents. Our study investigated the role of vagal afferents in anxiety-like behavior consistently in both sexes, allowing for the investigation of sex differences. We used free-cycling intact females, as our group recently found no impact of the estrous phase on anxiety-like parameters in the open field, elevated plus maze, or acoustic startle response tests (48). In this study, we found little to no differences between sexes in the effects of the feeding status or acute chemogenetic manipulation of vagal afferents on anxiety-like behavior. However, chronic interruption of gastrointestinal vagal feedback reduced anxiety-like behavior in male rats but not in female rats. In males, this anxiolytic phenotype associates with transcriptional changes in the CeA consistent with a potentiation of GABAergic signaling. These changes were not present (or greatly attenuated) in female rats, providing a molecular basis for the sexually dimorphic effect of CCK-SAP on anxiety-like behavior.

Altogether, our findings indicate that gut-innervating vagal afferents are necessary and sufficient for the acute metabolic tuning of anxiety in both male and female rats but strongly suggest that female baseline anxiety-like behavior is more resilient to long-term changes in vagal gastrointestinal feedback. This idea is supported by findings indicating that diet, and hence the consecutive chronic changes in gut-brain signaling, exert a sex-divergent effect on anxiety-like behavior (49,50).

### Conclusions

Our study unequivocally uncovers a role for vagal afferents from the gastrointestinal tract in anxiety modulation. We propose that these neurons tune anxiety-like behavior to meal-to-meal variations in the feeding status. Beyond this acute, physiological modulation of anxiety, we also found that a chronic lesion of gut-innervating vagal afferent neurons alters anxiety and anxiety-associated gene networks in the amygdala of male rats. This raises the novel question whether disruptions in gut-brain vagal feedback underlies the hypersensitivity and hypervigilance to somatic sensations seen in patients with anxiety disorders (2). If so, targeting vagal gut-brain signaling could normalize interoceptive feedback from the gastrointestinal tract in anxiety disorders.

## Supplementary Material

Supplemental figures
