# Supplementary material for: Neural Pathway for Gut Feelings: Vagal Interoceptive Feedback From the Gastrointestinal Tract Is a Critical Modulator of Anxiety-like Behavior
Source: Biol Psychiatry. Author manuscript; Available in PMC 2024 Sep 28. (PMC11438499; doi:10.1016/j.biopsych.2022.04.020)
Supplement: Supplemental figures [file NIHMS2020903-supplement-Supplemental_figures.pdf]

Neural Pathway for Gut Feelings: Vagal Interoceptive Feedback From the Gastrointestinal Tract Is a Critical Modulator of Anxiety-Like Behavior

Supplementary Information

Supplementary Methods

Animals and housing

For all behavioral and gene expression experiments, male and female Sprague-Dawley rats (n= 264, Charles River, 5 weeks old upon arrival) were pair housed with *ad libitum* access to water and standard laboratory chow (Envigo, 2018 Teklad Global 18% Protein Rodent Diet, 3.1 kcal/g) on 12 h:12 h light/dark cycle (lights on at 07:00). All procedures were approved by the Animal Welfare Committee of the University of Gothenburg and the Swedish Agriculture Ministry (Approvals #1/19 and 1/21). For polysynaptic tracing with Herpes Simplex Virus (HSV), male Wistar rats (n= 3, Envigo) were single housed with *ad libitum* access to water and standard laboratory chow (Envigo, 2018 Teklad Global 18% Protein Rodent Diet, 3.1 kcal/g) on 12 h:12 h light/dark cycle (lights on at 10:00). HSV study procedures were approved by IACUC and completed at University of Florida (Protocol #202110305).

Gastrointestinal injections

Gastrointestinal injections of the retrograde AAV AAVrg-pENN.AAV.hSyn.HI.eGFP-Cre.WPRE.SV40 (1.3E13 GC/mL, Addgene 105540-AAVrg) were done following a pattern consisting of 36 injection sites (stomach: 4 sites along the greater curvature, 4 sites on each of the ventral and dorsal fundus and body, 2 sites on each of the ventral and dorsal antrum, 4 around the circumference of the pylorus; duodenum: 4 sites around the circumference of the duodenum at 1 and 2 cm from the pylorus). This injection pattern was previously validated by

visualizing the spread of a green dye over the desired targeted gastrointestinal areas (Supp Fig 2G).

Anxiety tests

Rationale for the choice of behavioral tests

First, we used the open field and the elevated plus maze tests: both of these tests evaluate the animals' conflict between their drive to explore novel environments and their avoidance of open spaces (1, 2). In addition, we also used the food neophobia test, which evaluates another type of approach-avoidance conflict. In this test, rodents with high levels of innate anxiety typically take longer to start eating an unfamiliar food compared with less anxious rodents when the food is presented in a novel environment (3). Finally, we measured the acoustic startle response, which mirrors in rodents the heightened startle response observed in individuals with elevated baseline anxiety and anxiety disorders (2, 4). Importantly, the acoustic startle response is independent of the changes in locomotor activity which could confound approach-avoidance based tests. All tests were performed with a minimum of 48h recovery between tests.

Open field

The open field tests were conducted in two grey plexiglas arenas (100 x 100 cm) surrounded by 40 cm high walls. The arenas were placed in a dimly lit room, so that light intensity reached 35 lux in the center. For the purpose of scoring, a center zone (40 x 40 cm) was defined in the middle of the arenas. Rats were placed in a corner of the arenas and left to freely explore for 30 min. The level of anxiety was indexed by two variables: the distance moved in the center zone and the number of entries into the center zone. After the trial, the arenas were cleaned with 70% ethanol followed by warm water. The test was recorded with a camera placed above the arenas, allowing tracking and scoring of the rats' behavior with EthoVision 13 XT (Noldus Information Technology).

Elevated Plus Maze

The elevated plus maze consists in a grey plexiglas arena comprising four equally spaced arms (10 cm wide, 50 cm long) radiating out from a central square (10 cm) and elevated 60 cm above the floor. Two opposing arms are enclosed by walls (30 cm high; “closed arms”), whereas the other two arms are only guarded by a perimeter border of 1 mm (“open arms”). The maze was placed into a dimly lit room, so that light intensity reached 15 lux in the closed arms, and 55 lux in the open arms. Animals were placed in the central square of the maze and left to freely explore for 5 min. The entry of animals’ gravity center into any of the arms was automatically counted by the EthoVision tracking system. The levels of anxiety were indexed by two variables: the percentage of time spent in the open arms over the total test duration and the distance moved in the open arms over the total distance moved during the test. After the trial, the maze was cleaned with 70% ethanol followed by warm water. The test was recorded with a camera placed above the maze, allowing tracking and scoring of the rats’ behavior with EthoVision 13 XT (Noldus Information Technology).

Acoustic startle reflex

Rats were placed in a plexiglas cylinder (13 cm in diameter and 26 cm long for rats > 450 g and 9 cm in diameter and 200 cm long for rats < 450 g) connected to a piezoelectric accelerometer allowing for the recording of the amplitude of startle responses. Plexiglas cylinders were connected to and placed into bright ventilated chambers providing a background noise of 50 dB and delivering acoustic stimuli of 90, 105 or 120 dB (50 ms each). After 5 minutes of habituation to the chambers, the acoustic stimuli were presented in a randomized order (10 times for each intensity) with inter stimulus intervals ranging between 20 and 40 seconds using the SR Lab Software (San Diego Instruments, San Diego, USA). The peak amplitude response to each startle stimulus (in millivolts) was averaged across the 10 repetitions and used as the dependent measure. After each test, the cylinders were cleaned with 70% ethanol and warm water.

Food neophobia

The food neophobia test was conducted in a rectangular arena (37 x 21 cm) surrounded by transparent walls (19 cm high) and placed in a dimly lit room (50 lux in the center of the arena). The unfamiliar food (5 g of Peanut Butter, Skippy Extra smooth, Netherlands) was available in a vertical circular plate (diameter 10 cm) fixed to a wall of the arena. The latency to eat was defined as the time between the start of the test and the moment a rat started eating continuously for 3 seconds. The animal was then immediately removed from the arena. The same test, along with treatment (cohorts 2-4), was performed on two consecutive days to test whether nephobia persists over two exposures. The unfamiliar food and corresponding plates were replaced with new ones after each rat.

Adjustment of anxiety-like behavior variables for locomotor activity or hunger

Analyses of covariance (ANCOVA) were conducted using the rstatix package (v. 0.4.0) for R. For variables collected in the open field and elevated plus maze, the total distance moved during the test was used as a covariate. For the latency to eat a novel food in the neophobia test, the chow intake within 30 minutes following the end of the test was used as a covariate. In all graphs and figure legends, the main treatment effect is reported after adjustment for these covariates. Graphs show estimate marginal means calculated with the emmeans package (v. 1.6.1) for R .

Food intake measurements

Food intake was measured in SAP and CCK-SAP rats to evaluate completeness of gastrointestinal vagal deafferentation. The test was performed after all behavioral assessments to ensure that deafferentation was complete throughout the experiments. Briefly, rats were fasted for 15 hours (12 hours dark phase + 3 hours in the light phase) before they received an ip injection of 4 µg/kg CCK-8 (Tocris #1166) or vehicle (saline) in a randomized

cross-over within-subject design. Food was returned immediately and manually measured after 30 minutes. CCK-SAP rats that showed more than a 40% reduction in 30-min food intake after CCK compared to Veh injections were removed from the analysis (n= 3 males; 2 females). The effect of CNO injection on food intake was measured in hm3dGq-, hm4dGi- and non-DREADD-expressing rat cohorts. Briefly, rats were fasted for 6h into the light phase and chow was returned at dark onset. Rats received an injection of vehicle or CNO (2 mg/kg ip) 15 (hm4dGi cohort) or 30 (hm3dGq and non-Dreadd cohorts) minutes before dark onset in a within-subject counterbalanced designed. Food was then manually weighed after 1 and 24h.

Central amygdala cannulation and drug delivery

After anesthesia, rats were placed in a stereotaxic apparatus and implanted bilaterally with a 26-gauge guide cannula (Plastics One) using the following coordinates: 2.8 mm posterior to bregma, ± 4.8 mm from the midline, and 6.6 mm ventral to the surface of the skull, with injector aimed 8.6 mm ventral to skull. After a week of recovery, rats were habituated to the procedure for central injections. On experimental days, the GABAa receptor antagonist bicuculline (1(S),9(R)-(−)-Bicuculline methiodide, Sigma, 100 pmol in 0.2 uL per side, delivered over one minute) or vehicle (aCSF, Tocris) was administered 15 minutes prior to testing. The injection site through the amygdala guide cannula was verified post mortem, by microinjection of India ink at the same microinjection volume (0.2 μl) used in the study.

Measurement of gastrointestinal contents

After anesthesia, the stomach and proximal intestine were clamped at 0, 5, 10, 20 and 30 cm distal to the pyloric sphincter to prevent further movement of food down the gastrointestinal tract. Gastrointestinal segments were dissected out and weighed before and after removing their inner contents, allowing for the determination of contents’ weights. To account for the different sizes of gastrointestinal segments, stomach and intestinal contents are expressed as relative increase in tissue weight.

Terminal procedures

All rats were deeply anesthetized with an injection of ketamine (75 mg/kg ip; Ketaminol, Intervet) and xylazine (10 mg/kg; Rompun, Bayer) after the end of planned behavioral experiments, or 5 days after the nodose ganglia injections of Herpes Simplex Virus.

Nodose ganglia collection

Rats were perfused intracardially with ice-cold oxygenated aCSF for 3 minutes. The salivary glands, lymph nodes, sternohyoid, and omohyoid muscles were quickly dissected, and the vagus nerve was separated from the carotid arteries. The vagus nerve was cut 1 cm caudal to the nodose ganglia and both nodose ganglia were gently extracted. Nodose ganglia were separated from surrounding tissue in ice-cold PBS.

Brain collection

For gene expression studies, rats were fasted for one hour, during the light phase, to eliminate acute effects of feeding, then perfused intracardially with ice-cold oxygenated aCSF for 3 minutes and the brain was immediately extracted and frozen in isopentane on dry ice. To visualize the spread of Herpes Simplex Virus, rats were first intracardially perfused with PBS followed by 4% paraformaldehyde in PBS (pH = 7.4) before decapitation.

Histological procedures

Tissue preparation

Nodose ganglia were fixed by immersion for 2h in 4% paraformaldehyde in PBS and switched to 25% sucrose in PBS at 4˚C until further processing. Brains were post-fixed overnight in 4% paraformaldehyde in PBS and kept in 25% sucrose in PBS for at least 48h before downstream applications. Both tissues were cut in a cryostat (Leica CM3050S; nodose ganglia 14 - 18 um; brain 35 um) and collected on Superfrost Plus slides.

Immunostainings

Primary antibodies used in nodose ganglia sections are: chicken anti-GFP (Abcam, ab13970, 1:200), rabbit anti mCherry (Abcam, ab167453, 1:200). Primary antibodies used in brain sections are: goat anti-GFP (Abcam, ab125096, 1:1000) and mouse anti-mCherry (Abcam, ab6673, 1:500). Secondary antibodies used in nodose ganglia sections are: donkey anti-rabbit AF568 (Invitrogen, 2207536, 1:400), goat anti-chicken AF488 (Abcam 150169 1:400). Secondary antibodies used in brain sections are: donkey anti-goat AF488 (Jackson ImmunoResearch Laboratories, # 705-545-147, 1:1000), donkey anti-mouse AF594 (Invitrogen, #A-21203, 1:500)

Image acquisition

Nodose ganglia images were acquired using LSM700 Zeiss confocal microscope and processed using Zen lite software. Brain images were acquired with a Keyence BZ-X810 microscope with BZ-X Analyzer software.

Gene expression procedures

Gene expression in nodose ganglia with qPCR

In CCK-SAP injected rats and corresponding controls, RNA from nodose ganglia was extracted using Trizol (Invitrogen) according to the manufacturer’s protocol and quantified with Nanodrop (Thermofisher). Reverse transcription was performed using iScript cDNA kit (Biorad). qPCR was performed on a QuantStudio 7 Flex Real-Time PCR System (Applied Biosystems) using TaqMan assays (Thermofisher: Gapdh Rn01775763_g1; Cckar Rn00562164_m1). Results were quantified using the 2-ddCtt method.

RNA sequencing from brain micropunches

Frozen aCSF-perfused brains from male CCK-SAP and their corresponding controls were cut in 50 um coronal slices using a cryostat (Leica CM3050S). The CeA and BnST were

microdissected using biopsy bunches (Integra Miltex, 1 mm diameter), collected into RNAse free tubes and kept at -80˚C until further processing. Both areas were identified anatomically and using stereotaxic coordinates according to “The Rat Brain” (Paxinos and Watson, 5th edition); CeA Bregma -3.24 to -1.56 mm and BnST Bregma -0.48 to +0.36 mm.

RNA from brain micropunches was extracted using Trizol (Invitrogen) according to the manufacturer’s protocol. Before RNA sequencing, the integrity of mRNA from brain micropunches was verified using the Agilent 2100 bioanalyzer. Samples with RNA integrity number (RIN) above 8 were further processed and included in the analysis.

Library preparation, mRNA sequencing, quality controls and mapping were performed by Novogene (China). NEB Next® Ultra RNA Library Prep Kit for Illumina was used to produce library constructs. Briefly, mRNA was purified from total RNA using poly-T oligo-attached magnetic beads. After fragmentation, the first strand cDNA was synthesized using random hexamer primers followed by the second strand cDNA synthesis. The library was ready after end repair, A-tailing, adapter ligation, and size selection. After amplification and purification, insert size of the library was validated on an Agilent 2100 and quantified using quantitative PCR. Libraries were then sequenced on Illumina NovaSeq 6000 S4 flowcell with PE150. All reads were quality-checked and reads with adaptor contamination, uncertain nucleotides (more than 10% of the read contains “N”) or low quality nucleotides (base quality less than 20 constitute more than 50% of the read) were discarded. Reads were subsequently aligned with HISAT2 using the rat genome build and annotation from Ensembl (Rnor_6.0).

RNA sequencing data from the CeA and BnST of CCK-SAP and SAP male rats is deposited at the European Nucleotide Archive (PRJEB48979).

Analysis of gene expression from RNA sequencing

Differential gene expression was computed for pairwise comparisons (CCK-SAP versus SAP) using the Bioconductor package *DESeq2* for R (5) on the basis of read counts. Principal component analysis was computed using the function plotPCA of *DESeq2.* Heatmaps show the relative expression (z-score) of significant DEGs in SAP and CCK-SAP rats.

Gene-disease association

Gene-disease associations were computed using the Bioconductor package *CTDquerier* for R (6), which retrieves data from the Comparative Toxicogenomics database (CTD; http://ctdbase.org/ (7)). Briefly, for each brain area, the association between the differentially-expressed genes and “Anxiety disorders” was assessed on the basis of curated and predicted associations reported in the CTD. We then tested whether the lists of DEGs show an overrepresentation of genes associated with “Anxiety disorders” compared to all detected genes in each region, using the *enrich* function of the *CTDquerier* package for R (6).

Identification of biological processes from transcriptomic data

Biological processes affected by gastrointestinal vagal deafferentation in the CeA were identified by Gene Ontology (GO) enrichment analysis. We used the *enrichGO* function from the *ClusterProfiler* package for R (8) to return enriched GO categories (subontology “Biological Processes”) among the identified DEGs (Bonferroni-Holm adjusted $p <= 0.05$, q-value $<= 0.05$). The biological process “Metanephros development” (4 associated genes, among which one uniquely associated to this process) was removed from the analysis.

Statistical approach to treatment and sex-specific effects

We first examined whether the effect of the treatment of interest differs by sex using the ANOVA sex x treatment interaction term. When sex did not interact with the treatment variable, we reported the main effect of the treatment. Therefore, most figures and legends report only one p-value, independently of sex. When sex interacted with the treatment variable, we reported the interaction term and compared relevant groups using Holm-adjusted comparisons. All statistical tests were generated with R (v. 3.6.1) and scripts are available upon request.

Supplementary Figures

Supplementary Figure 1:

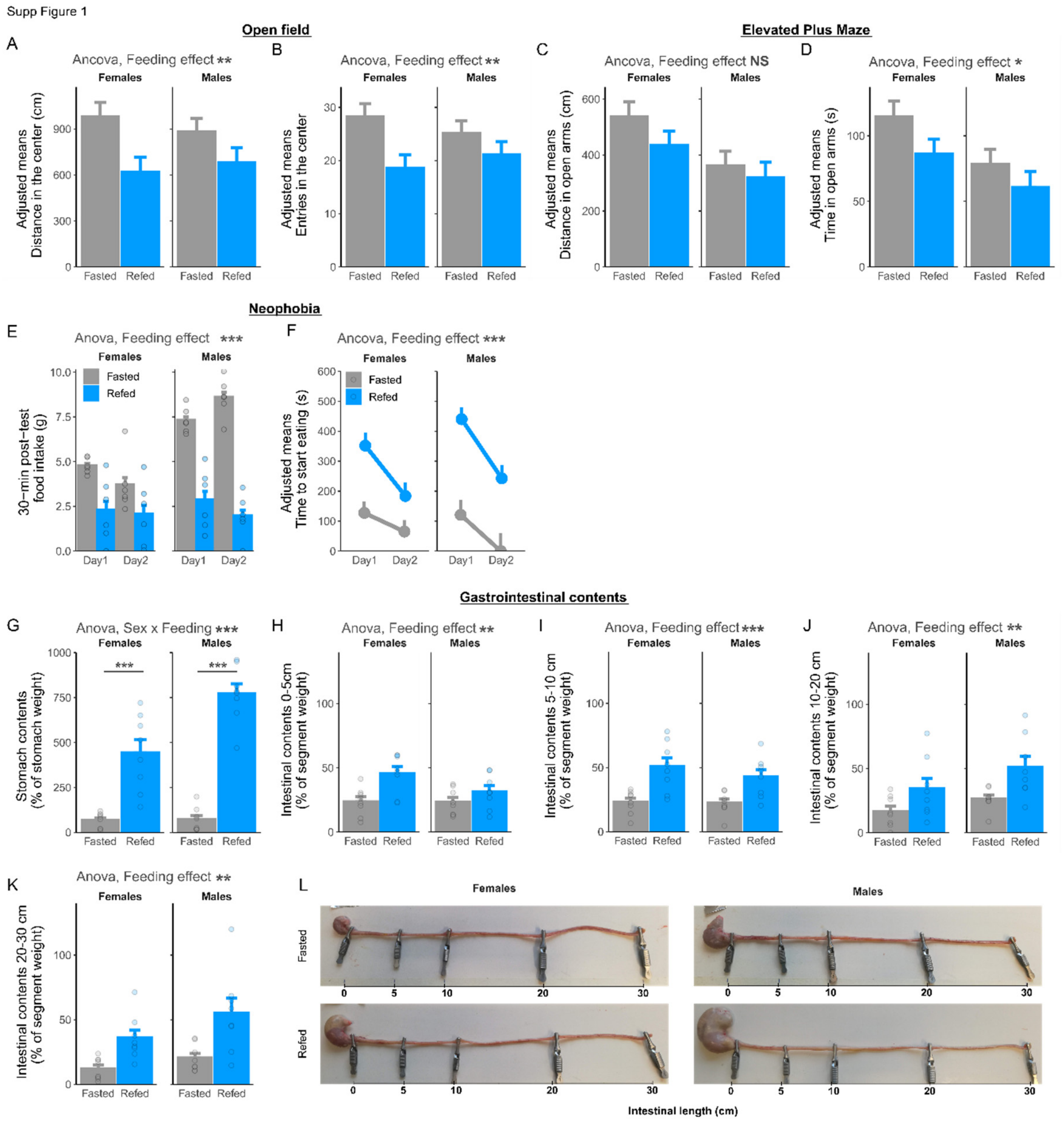

A. Mean distance and **B.** number of entries in the center zone of an open field in fasted and refed rats adjusted for total distance. Refeeding significantly decreases distance (ANCOVA, $F[1,42] = 10.21$, $p = 0.003$) and number of entries (ANCOVA, $F[1,42] = 8.91$, $p = 0.005$) despite adding total distance as a covariate.

C. Mean distance and **D.** time in the open arms of an elevated plus maze in fasted and refed rats adjusted for total distance. Mean time (ANCOVA, $F[1,44] = 4.15$, $p = 0.048$), but not

distance (ANCOVA, $F[1,44] = 1.99$, $p = 0.16$), significantly differs between fed and fasted rats after addition of total distance as a covariate.

E. Food intake during the 30 minutes following the end of the neophobia test (ANOVA, Feeding effect, $F[1,44] = 180.53$, $p < 0.001$)

F. Mean time to start eating a novel food in fasted and fed rats, adjusted for hunger (measured as food intake in the 30 minutes following the end of the neophobia test). Refeeding increases the time to start eating a novel food compared to fasting after addition of hunger as a covariate (ANCOVA, $F[1,90] = 20.11$, $p = 2.15.10^{-5}$).

G. 1h *ad libitum* refeeding increases stomach contents in both males and females, but the extent of this increase depends on sex (ANOVA, Sex x Feeding status interaction, $F[1,28] = 11.00$, $p = 0.003$; Holm-adjusted comparisons, Females $p = 7.38.10^{-6}$, Males $p = 6.54.10^{-11}$).

H-K. 1h *ad libitum* increases the contents of all intestinal segments (0-5 cm: ANOVA, $F[1,29] = 10.95$, $p = 0.003$; 5-10 cm: ANOVA, $F[1,29] = 23.88$, $p = 3.48.10^{-5}$; 10-20 cm: ANOVA, $F[1,29] = 10.94$, $p = 0.003$; 20-30 cm: ANOVA, $F[1,29] = 18.41$, $p =1.81.10^{-4}$)

L. Representative pictures of stomach and intestine (0-30 cm) after fasting or 1h *ad libitum* refeeding.

Supplementary Figure 2:

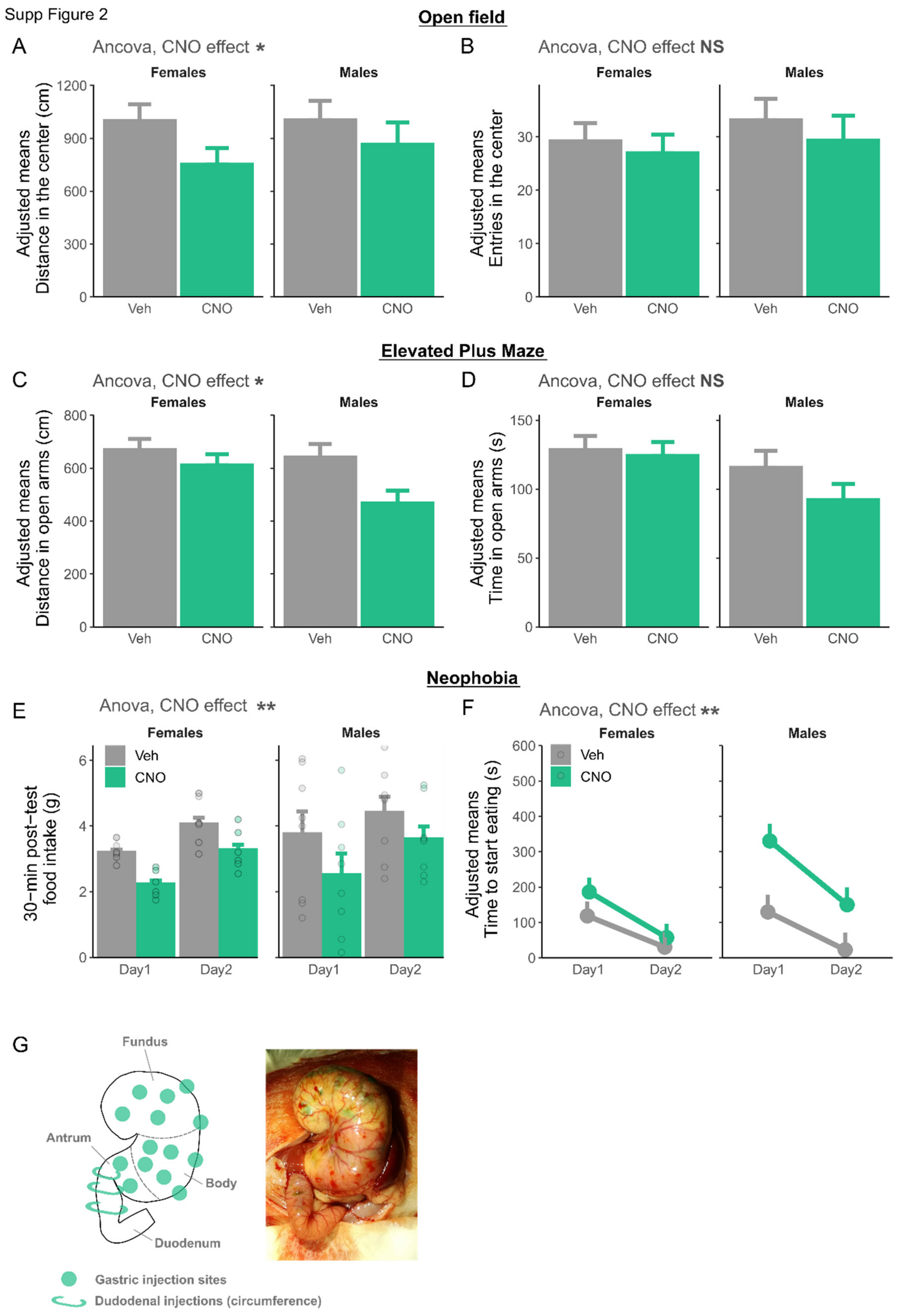

A. Mean distance in the center zone of an open field is reduced in CNO-injected compared to vehicle-injected rats after adjustment for total distance (ANCOVA, $F[1,31] = 1.13$, $p = 0.05$).

B. Number of entries in the center zone of an open field remain similar in Veh- and CNO-injected rats after adjustment for total distance (ANCOVA, $F[1,31] = 0.59$, $p = 0.45$).

C. Mean distance in the open arms of an elevated plus maze is reduced in CNO-injected compared to Veh-injected rats after adjustment for total distance (ANCOVA, $F[1,35] = 5.15$, $p = 0.03$).

D. Mean time in the open arms of an elevated plus maze remain similar in Veh- and CNO-injected rats after adjustment for total distance (ANCOVA, $F[1,35] = 1.19$, $p = 0.28$).

E. Food intake during the 30 minutes following the end of the neophobia test (ANOVA, CNO effect, $F[1,35] = 9.63$, $p = 0.004$).

F. CNO injection increases the time to start eating a novel food compared to Veh injection after addition of hunger (measured as food intake in the 30 minutes following the end of the neophobia test) as a covariate (ANCOVA, $F[1,72] = 8.02$, $p = 0.006$).

G. Injection pattern used to administer retrograde AAVs to the stomach and duodenum (left) with a representative picture of the stomach and duodenum of a male rat after injection of a green dye following this pattern (right).

Supplementary Figure 3:

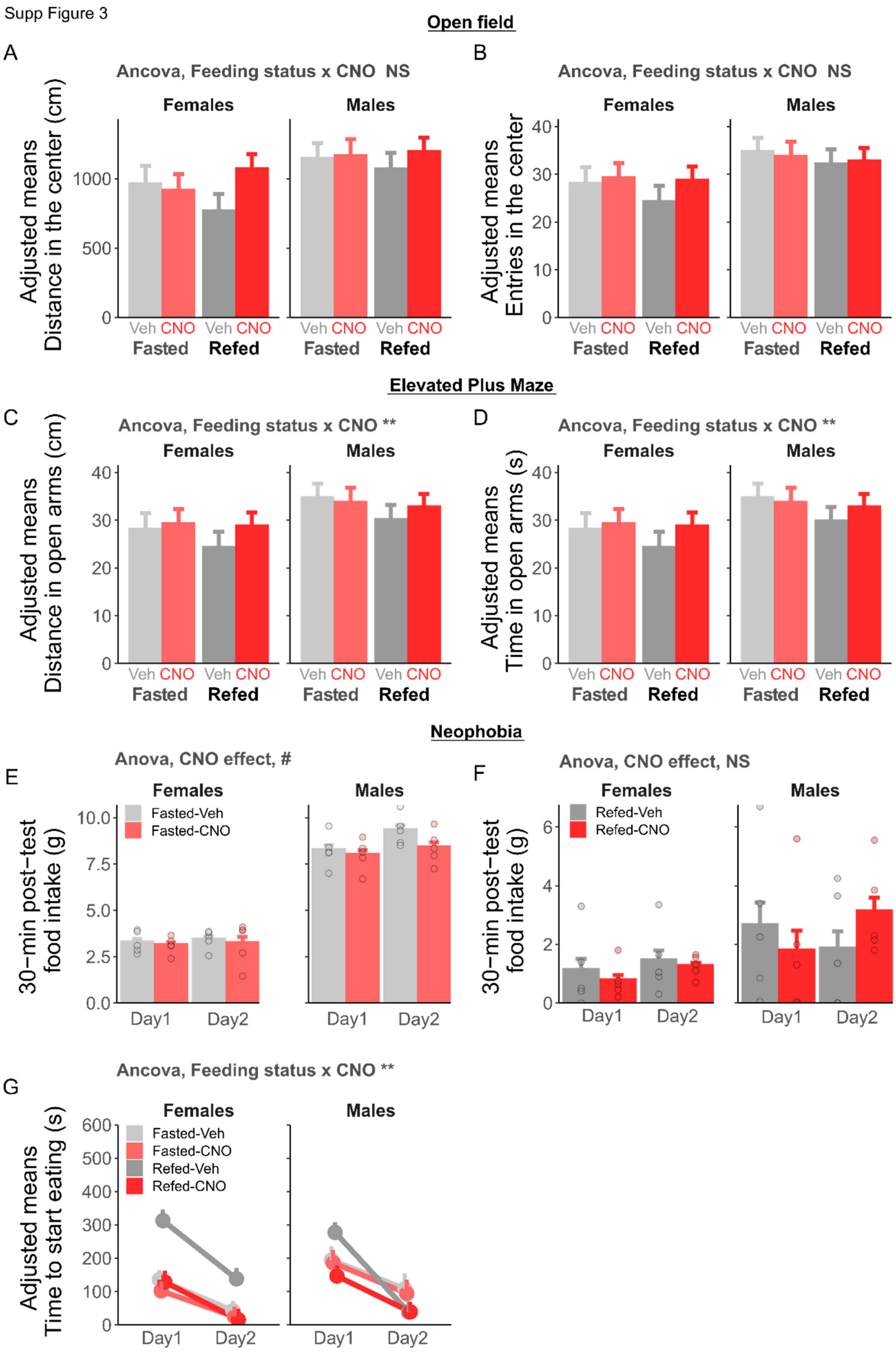

A. Mean distance in the center zone of an open field does not differ between CNO-injected and vehicle-injected rats after adjustment for total distance, independent of the feeding status (ANCOVA, $F[1,67] = 2.01$, $p = 0.16$).

B. Number of entries in the center zone of an open field does not differ between CNO-injected and vehicle-injected rats after adjustment for total distance, independent of the feeding status (ANCOVA, $F[1,67] = 0.41$, $p = 0.53$).

C. The effects of CNO on the mean distance in the open arms of an elevated plus maze depend on the feeding status after adjustment for the total distance moved (ANCOVA, $F[1,74] = 8.61$, $p = 0.004$).

D. The effects of CNO on the mean time spent in the open arms of an elevated plus maze depend on the feeding status after adjustment for the total distance moved (ANCOVA, $F[1,74] = 10.17$, $p = 0.002$).

E. Food intake during the 30 minutes following the end of the neophobia test in fasted animals (ANOVA, CNO effect, $F[1,36] = 3.65$, $p = 0.064$).

F. Food intake during the 30 minutes following the end of the neophobia test in refed rats (ANOVA, CNO effect, $F[1,36] = 0.004$, $p = 0.95$).

G. CNO inhibition decreases the time to start eating a novel food in a feeding status-dependent manner after adjustment for hunger (measured as food intake in the 30 minutes following the end of the neophobia test) as a covariate (ANCOVA, feeding status * CNO, $F[1,150] = 10.17$, $p = 0.002$).

Supplementary Figure 4:

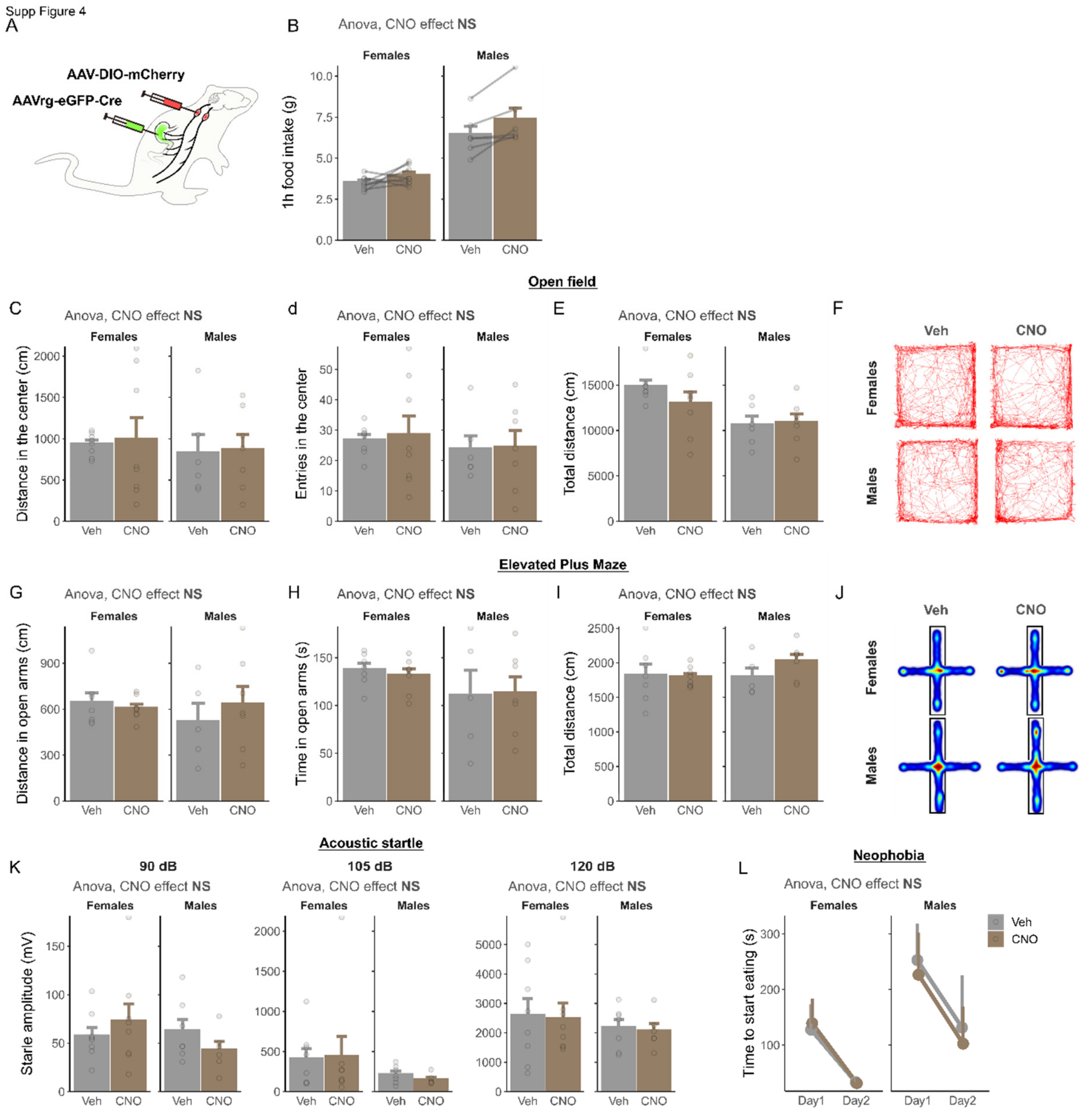

A. Dual viral injection strategy used to generate rats expressing eGFP and mCherry, but no hm3d/hm4D into gastrointestinal vagal afferents.

B. 1h food intake does not differ between Veh- and CNO-injected rats (ANOVA, $F[1,25] = 2.87$, $p = 0.13$).

C. Distance (ANOVA, $F[1,26] = 0.07$, $p = 0.80$).and **D.** number of entries (ANOVA, $F[1,26] = 0.06$, $p = 0.81$) in the center of an open field do not differ between Veh- and CNO-injected rats.

E. The total distance moved in an open field is not significantly changed by CNO injection (ANOVA, $F[1,26] = 0.87$, $p = 0.36$).

F. Representative traces of Veh- or CNO-injected rats during a 30-min open field test.

G. Distance (ANOVA, $F[1,24] = 0.11$, $p = 0.74$) and **H.** Time (ANOVA, $F[1,24] = 0.025$, $p = 0.88$) spent in the open arms of an elevated plus do not differ between Veh- and CNO-injected rats.

I. The total distance moved in an elevated plus maze is not significantly changed by CNO injection (ANOVA, $F[1,24] = 0.56$, $p = 0.46$).

J. Average heatmaps of Veh- or CNO-injected rats during a 5-min elevated plus maze test.

K. Startle amplitude in response to acoustic stimuli is not changed by CNO injection (ANOVA 90dB: $F[1,26] = 0.00003$, $p = 0.99$; 105 dB: $F[1,26] = 0.004$, $p= 0.95$; 120 dB $F[1,26] = 0.053$, $p = 0.82$).

L. Time to start eating a novel food does not differ between Veh- and CNO-injected rats (ANOVA, $F[1,53] = 0.07$, $p = 0.80$).

Supplementary Figure 5:

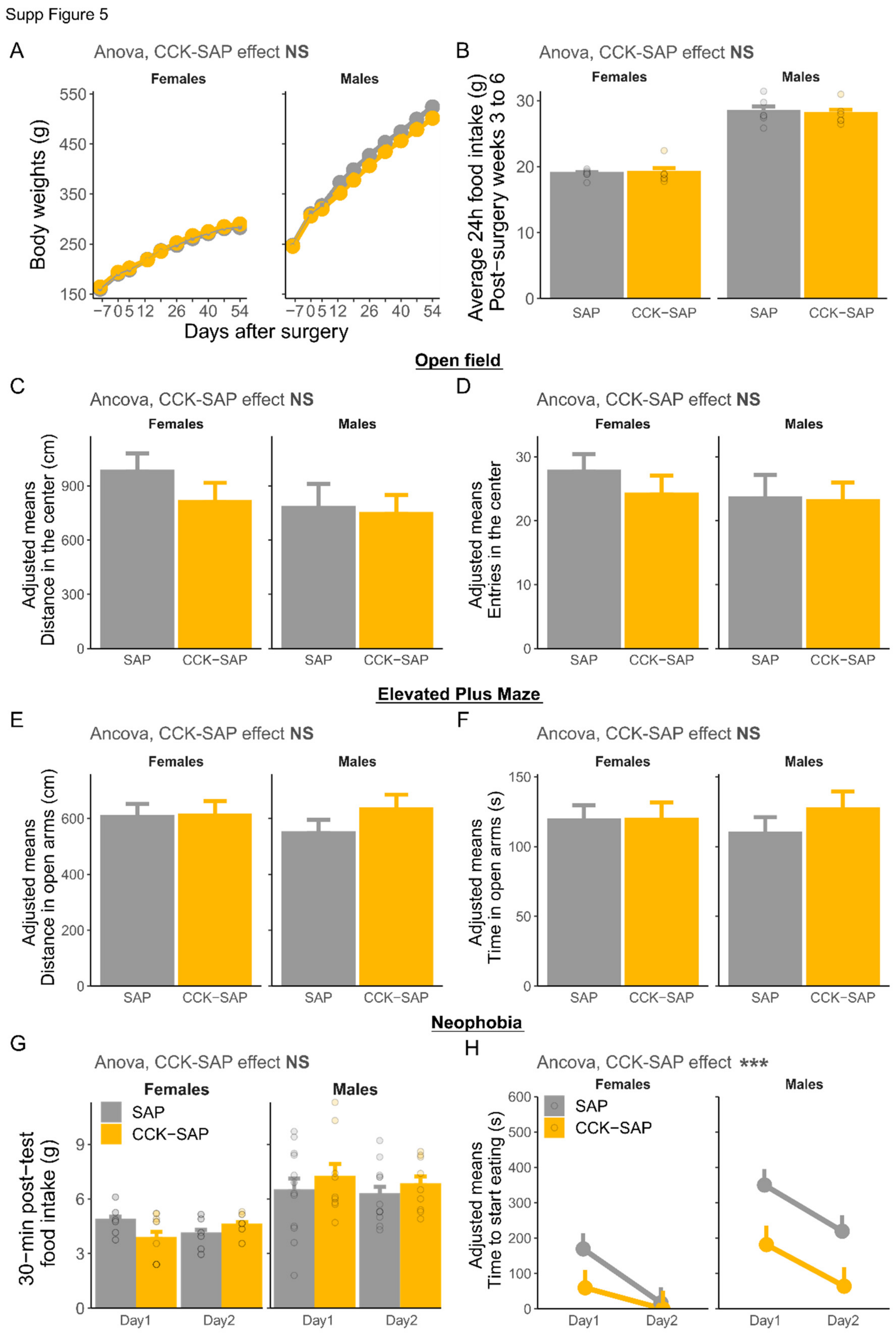

A. Body weights (ANOVA, $F[1,39] = 0.692$, $p = 0.41$) and **B.** 24h food intake (average of two data points per week through weeks 3 to 6 post-surgery; ANOVA, $F[1,20] = 0.019$, $p = 0.89$) do not differ between SAP and CCK-SAP rats.

C. Mean distance (ANCOVA, $F[1,37] = 1.24$, $p = 0.27$) and **D.** number of entries (ANCOVA, $F[1,37] = 0.68$, $p = 0.42$) in the center zone of an open field are not different between SAP an CCK-SAP rats after adjustment for total distance.

E. Mean distance (ANCOVA, $F[1,39] = 0.79$, $p = 0.38$) and **F.** time (ANCOVA, $F[1,39] = 0.54$, $p = 0.47$) in the open arms of an elevated plus maze are not different between SAP an CCK-SAP rats after adjustment for total distance.

G Food intake during the 30 minutes following the end of the neophobia test (ANOVA, CCK-SAP effect, $F[1,39] = 0.19$, $p = 0.66$).

H. CCK-SAP decreases the time to start eating a novel food compared to SAP after addition of hunger (measured as food intake in the 30 minutes following the end of the neophobia test) as a covariate (ANCOVA, $F[1,80] = 11.75$, $p = 0.001$).

Supplementary Figure 6:

Supp Figure 6

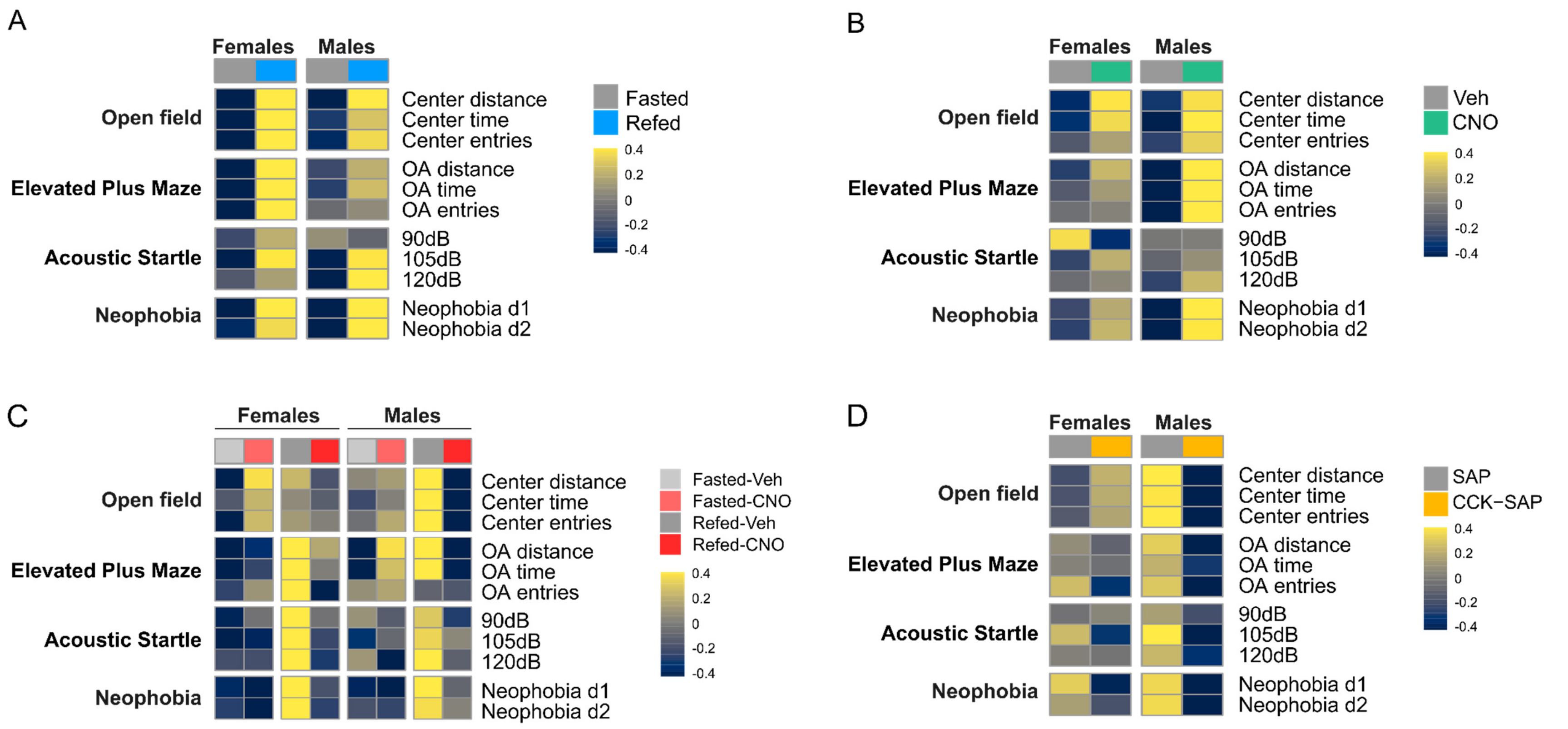

Summary heatmaps of surrogate markers of anxiety-like behavior in four rat cohorts (sex-and cohort-specific z-scores)

A. Overnight fasted or 1h refed rats (Cohort 1)

B. Chemogenetic activation of gut-projecting vagal afferents (Cohort 2)

C. Chemogenetic inhibition of gut-projecting vagal afferents in overnight fasted or 1h refed rats (Cohort 3)

D. Gastrointestinal vagal deafferentation by CCK-SAP (Cohort 5)

Supplementary Figure 7:

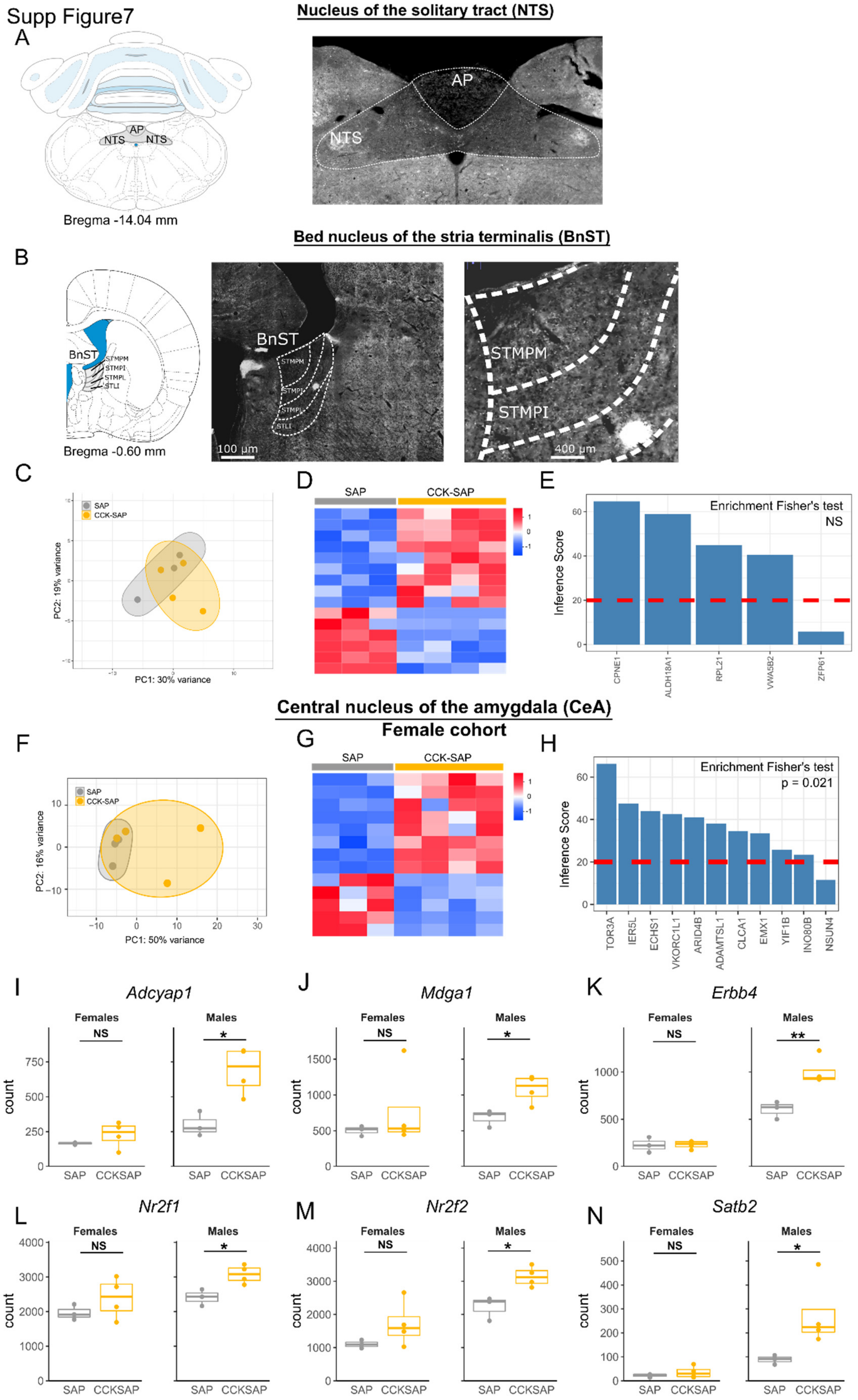

A. Representative picture of HSV-129 labelling in the nucleus of the solitary tract (NTS) five days after HSV-129 injection into the nodose ganglia (20X magnification; AP area postrema)

B. Representative picture of HSV-129 labelling in the BnST five days after HSV-129 injection into the nodose ganglia (STMPM bed nucleus of the stria terminalis, medial division, posteromedial part; STMPI bed nucleus of the stria terminalis, medial division, posterointermediate part; STMPL bed nucleus of the stria terminalis, medial division, posterolateral part ; STLI bed nucleus of the stria terminalis, lateral division, intermediate part)

C. Principal component analysis plot of BnST gene expression profiles in CCK-SAP and control rats

D. Heatmaps of gene expression (z-score) for significant differentially-expressed genes (DEGs) in the BnST of CCK-SAP compared to control rats (adjusted $p < 0.05$)

E. Inference scores of DEGs in the BnST associated with "Anxiety disorders" according to the Comparative Toxicogenomics Database (CTD).

F. Principal component analysis plot of CeA gene expression profiles in CCK-SAP and control female rats.

G. Heatmaps of gene expression (z-score) for significant differentially-expressed genes (DEGs) in the CeA of CCK-SAP compared to control female rats (adjusted $p < 0.05$)

H. Inference scores of DEGs in the CeA of female rats associated with "Anxiety disorders" according to the Comparative Toxicogenomics Database (CTD).

I-N. CeA expression (normalized counts) of genes previously identified as DEG in the CeA of male CCK-SAP rats and associated with the regulation and maintenance of GABAergic synapses (Bonferroni adjusted p-values: *Adcyap1*, females $p = 0.27$, males $p = 0.013$; *Mdga1*, females $p = 0.39$, males $p = 0.021$; *Erbb4*, females $p = 0.97$, males $p = 0.0072$; *Nr2f1*, females $p = 0.26$, males $p = 0.020$; *Nr2f2*, females $p = 0.17$ males $p = 0.024$; *Satb2*, females $p = 0.37$, males $p = 0.044$.)

Supplementary Tables

Supplementary Table 1: Differentially-expressed genes (DEGs) in the central amygdala (CeA) after CCK-SAP compared to SAP (male cohort).

Gene names	log2(FoldChange)	adjusted p-values
Cttn	1.53	1.81e-30
LOC100911727	3.1	9.44e-08
Ttr	11.19	3.45e-07
Rps9l1	-1.51	5.16e-05
LOC103690028	-4.09	5.7e-05
F5	8.79	0.000625
Nol8	-2.29	0.00067
Fut9	1.59	0.000821
Map3k2	0.76	0.00129
Adcyap1	1.2	0.00129
Cryab	0.53	0.00135
Col23a1	1.36	0.00135
Erbb4	0.74	0.00135
Kcnj13	8.08	0.00136
LOC100910882	2.17	0.00173
Mis18a	-3.53	0.00197
Fhad1	1.61	0.00269
Cldn2	9.13	0.00289
Satb2	1.66	0.0029
Ccdc142	-3.01	0.00467
Rasgrp4	-0.88	0.00643
Rsl1d1l1	-2.61	0.00651
Pdyn	-0.78	0.00746

Col1a2	1.2	0.00931
Mfrp	5.92	0.00931
Strip2	-0.35	0.00931
Pkhd1l1	3.05	0.00959
Dmrt3	7.35	0.00959
AABR07058985.1	-3.16	0.00959
Six3	-0.52	0.00959
AABR07027870.1	-0.33	0.00959
Esm1	7.39	0.00977
Ttc23	0.84	0.0135
Gnal	-0.53	0.0142
Scai	0.89	0.0147
Bhmt	4.52	0.015
Slco1a2	7.07	0.0168
Lipm	6.19	0.0172
Eea1	0.32	0.0175
AABR07049499.1	6.55	0.0175
B4gat1	-3.24	0.0175
Mal	0.54	0.019
LOC103690067	2.43	0.019
Clic6	6.13	0.0207
AABR07000382.1	-4	0.0207
Pla2g5	5.43	0.0222
Pde7b	-0.5	0.0228
Slc4a5	5.56	0.0258
Cnmd	4.68	0.0258
Mab21l3	5.19	0.0258

Car13	3.26	0.0258
RGD1560775	-1.36	0.0258
Cemip	0.47	0.0285
Tet1	0.93	0.0285
Nr2f2	0.5	0.0286
Penk	-0.62	0.0289
Phip	0.43	0.0324
Ogdhl	-0.29	0.0324
Akr1b1	-0.3	0.0367
Zdhhc22	-0.46	0.0376
LOC108348049	-3.48	0.0405
Mdga1	0.67	0.0406
Nfs1	-4.31	0.0496
AABR07072264.3	0.64	0.0496

Supplementary Table 2: Differentially-expressed genes (DEGs) in the central amygdala (CeA) after CCK-SAP compared to SAP (female cohort).

Gene names	log2(FoldChange)	adjusted p-values
Echs1	-3.63	2.02e-23
Tor3a	0.80	0.0030
Vkorc1l1	3.04	0.0030
Yif1b	2.65	0.0060
Clca1	0.93	0.0060
LOC100909555	-1.67	0.0065
Adamtsl1	1.22	0.010
Emx1	2.75	0.019
Ier5l	0.67	0.031
Atp5f1	-1.65	0.031
Ino80b	-2.50	0.031
Arid4b	1.41	0.038
Nsun4	-3.04	0.044

Supplementary Table 3: Differentially-expressed genes (DEGs) in the bed nucleus of the stria terminals (BnST) after CCK-SAP compared to SAP (male cohort).

Gene names	log2(FoldChange)	adjusted p-values
LOC108348080	-3.71	2.45e-25
Rsl1d1l1	3.04	4.15e-15
LOC103690067	2.11	1.46e-05
AABR07066693.1	0.95	1.46e-05
LOC100909505	1.8	0.000111
Vwa5b2	-0.31	0.00079
LOC100910308	-2.85	0.00203
Zfp61	-0.27	0.00808
LOC685716	-6.64	0.0111
Rpl21	0.32	0.0183
LOC103689986	3.58	0.0224
AC134224.2	-0.34	0.0224
Cpne1	1.57	0.0332
LOC103692173	2.42	0.0378
Aldh18a1	2.61	0.043

Supplementary References:

1. Belzung C, Griebel G (2001): Measuring normal and pathological anxiety-like behaviour in mice: a review. *Behav Brain Res*. 125:141-149.
2. Cryan JF, Holmes A (2005): The ascent of mouse: advances in modelling human depression and anxiety. *Nat Rev Drug Discov*. 4:775-790.
3. Merali Z, Levac C, Anisman H (2003): Validation of a simple, ethologically relevant paradigm for assessing anxiety in mice. *Biol Psychiatry*. 54:552-565.
4. Poli E, Angrilli A (2015): Greater general startle reflex is associated with greater anxiety levels: a correlational study on 111 young women. *Front Behav Neurosci*. 9:10.
5. Love MI, Huber W, Anders S (2014): Moderated estimation of fold change and dispersion for RNA-seq data with DESeq2. *Genome Biol*. 15:550.
6. Hernandez-Ferrer C, Gonzalez JR (2018): CTDquerier: a bioconductor R package for Comparative Toxicogenomics DatabaseTM data extraction, visualization and enrichment of environmental and toxicological studies. *Bioinformatics*. 34:3235-3237.
7. Davis AP, Grondin CJ, Johnson RJ, Sciaky D, Wiegers J, Wiegers TC, et al. (2021): Comparative Toxicogenomics Database (CTD): update 2021. *Nucleic Acids Res*. 49:D1138-D1143.
8. Wu T, Hu E, Xu S, Chen M, Guo P, Dai Z, et al. (2021): clusterProfiler 4.0: A universal enrichment tool for interpreting omics data. *Innovation (N Y)*. 2:100141.
